# Molecular Cloning and Functional Characterization of the Dehydrin (*IpDHN*) Gene From *Ipomoea pes-caprae*

**DOI:** 10.3389/fpls.2018.01454

**Published:** 2018-10-11

**Authors:** Hui Zhang, Jiexuan Zheng, Huaxiang Su, Kuaifei Xia, Shuguang Jian, Mei Zhang

**Affiliations:** ^1^Key Laboratory of South China Agricultural Plant Molecular Analysis and Genetic Improvement, South China Botanical Garden, Chinese Academy of Sciences, Guangzhou, China; ^2^College of Life Sciences, University of Chinese Academy of Sciences, Beijing, China; ^3^Key Laboratory of Applied Botany, South China Botanical Garden, Chinese Academy of Sciences, Guangzhou, China

**Keywords:** *Ipomoea pes-caprae* L., *dehydrin*, salt, drought, promoter

## Abstract

Dehydrin (DHN) genes can be rapidly induced to offset water deficit stresses in plants. Here, we reported on a dehydrin gene (*IpDHN*) related to salt tolerance isolated from *Ipomoea pes-caprae* L. (Convolvulaceae). The IpDHN protein shares a relatively high homology with Arabidopsis dehydrin ERD14 (At1g76180). IpDHN was shown to have a cytoplasmic localization pattern. Quantitative RT-PCR analyses indicated that *IpDHN* was differentially expressed in most organs of *I. pes-caprae* plants, and its expression level increased after salt, osmotic stress, oxidative stress, cold stress and ABA treatments. Analysis of the 974-bp promoter of *IpDHN* identified distinct *cis*-acting regulatory elements, including an MYB binding site (MBS), ABRE (ABA responding)-elements, Skn-1 motif, and TC-rich repeats. The induced expression of *IpDHN* in *Escherichia coli* indicated that IpDHN might be involved in salt, drought, osmotic, and oxidative stresses. We also generated transgenic Arabidopsis lines that over-expressed *IpDHN*. The transgenic Arabidopsis plants showed a significant enhancement in tolerance to salt/drought stresses, as well as less accumulation of hydrogen peroxide (H_2_O_2_) and the superoxide radical (O_2_^−^), accompanied by increasing activity of the antioxidant enzyme system *in vivo*. Under osmotic stresses, the overexpression of *IpDHN* in Arabidopsis can elevate the expression of ROS-related and stress-responsive genes and can improve the ROS-scavenging ability. Our results indicated that *IpDHN* is involved in cellular responses to salt and drought through a series of pleiotropic effects that are likely involved in ROS scavenging and therefore influence the physiological processes of microorganisms and plants exposed to many abiotic stresses.

## Introduction

The halophyte *Ipomoea pes-caprae* L., belonging to the Convolvulaceae family, is mainly distributed in the littoral region of tropical and subtropical areas worldwide (Miryeganeh et al., 2014) and has attracted attention due to its sand fixation, wind resistance, landscape greening, and ecological restoration abilities in coral islands and coastal zones (Ouyang et al., 2011). Throughout their whole life cycle, halophytes are subjected to abiotic stresses, including extreme salt damage, drought or dehydration, osmotic stress, and nutritional imbalance, depending on their habitats (Kumari et al., 2015). Understanding how halophytes respond to salinity or other abiotic stresses will provide us with genetic resources and tactics to develop salt-resistant crop varieties and therefore lay the basis for further land improvements and solonchak agriculture (Xu et al., 2016). High soil salinity and water deficiency are the major constraints on the growth and yield of many plant species (Pierik and Testerink, 2014). Halophytes, as the best potential resource for salt stress-tolerance genes, have provided a favorable platform for the characterization of salt-responding genes that can be adopted for improving salinity tolerance in crop plants through genetic engineering (Mishra and Tanna, 2017).

Dehydrins (DHNs) belong to LEA proteins, a large family of highly hydrophilic proteins, and are classified into Group II LEA proteins (Graether and Boddington, 2014). In addition to accumulating during the seed maturation process, LEA proteins also increase responding to abiotic stresses that could cause cellular dehydration or water-deficit, such as freezing, high salinity, and drought (Hand et al., 2011). Typically, most DHNs have three types of conserved sequence motifs, i.e., K- (EKKGIMDKIKEKLPG), Y- ([T/V]D[E/Q]YGNP), and S- (serine-track) motifs, among which K- is the core segment and sets one or more repeats, forming amphiphilic α-helixes located at the C-terminal end of the proteins (Cuevas-Velazquez et al., 2014). Accordingly, DHNs fall into five subclasses, including K_n_, K_n_S, Y_n_K_n_, SK_n_, and Y_n_SK_n_, mainly based on the replication of conserved K-, S-, and Y-segment motifs (Close, 1997). Further studies indicate that the K-segments are the functional core parts of DHNs that mediate cellular stress tolerance (Drira et al., 2013), bind to anionic phospholipid vesicles (Koag et al., 2009), or maintain enzyme activity (Yang et al., 2015).

Generally, DHNs are considered as stress proteins involved in multifunctional roles for plant protective reactions against dehydration by holding water molecules, binding transition metals to scavenge ROS, or binding to DNAs, proteins, sugars, or phospholipids to maintain biological activity (Liu et al., 2017). In addition, DHNs (or other LEA proteins) are known as IDPs and may serve as hub proteins and coordinate crosstalk with cellular signals and pathways involved in appropriate responses to stress conditions, or may be involved in the formation and stability of the cytoplasmic glassy state subjected to dehydration (Sun et al., 2013).

In recent years, numerous physiological studies have focused on the positive relationship between cellular accumulation of DHNs and increased plant tolerance to water-deficit stresses, including mainly cold, drought, and high salinity (Graether and Boddington, 2014). For example, an acidic SK_3_ DHN isolated from *Opuntia streptacantha* (Cactaceae) could lead to enhanced tolerance to freezing when overexpressed in Arabidopsis (Ochoa-Alfaro et al., 2012); wheat DHN’s (*DHN-5*) overexpression enhanced the salinity tolerance of transgenic Arabidopsis plants (Saibi et al., 2015); and *Saussurea involucrata*, a hardy dicotyledonous plant growing in alpine region, is capable of tolerating severe cold and arid conditions. Two DHN members, *SiDHN1* and *SiDHN2,* can elevate the cold tolerance of the transgenic overexpression of tobacco and showed an induced expression pattern when challenging cold stress in *S. involucrata* (Qiu et al., 2014; Guo et al., 2017). Oleaster is a typical drought-enduring plant in the Mediterranean area, and an oleaster DHN gene, named *OesDHN*, can improve drought tolerance when overexpressed in Arabidopsis (Chiappetta et al., 2015). *Prunus mume* ‘Beijingyudie’ is also a hardy plant and can withstand extremely cold temperatures even as low as −19°C, and the overexpression of four *P. mume dehydrins* in *Escherichia coli* and tobacco resulted in increased freezing resistance (Bao et al., 2017). Two *Hevea brasiliensis* DHN genes, *HbDHN1* and *HbDHN2*, could lead to a significant increase in the tolerance to salt, drought, and osmotic stresses when overexpressed in Arabidopsis (Cao et al., 2017). A DHN gene, *CdDHN4*, from bermudagrass (*Cynodon dactylon* L.), also showed an induced expression pattern when challenged by high/low temperatures, drought, salt and ABA (Lv et al., 2017). And also, *SbDhn1* from *Sorghum bicolor*, encoding an YSK2-type DHN, showed obvious transcript accumulation when subjected to high temperature and osmotic stress, and overexpression in tobacco plants led to a protective effect under high temperature and osmotic stress treatments (Halder et al., 2017). The above series of reports indicate that plant DHNs might be extensively involved in abiotic stresses, mainly water deficit.

Although there are numerous studies on the biological functions of DHNs in various plant species, the functional characterization of salt-responsive genes of *I. pes-caprae*, a unique halophyte, requires further investigation: DHNs from this species involved in salt and drought tolerance have not been reported until now. In our previous research, we constructed a cDNA library of *I. pes-caprae* and obtained a series of possible candidate salt-tolerance genes using the FOX gene hunting system, including a DHN gene (*IpSR38*, *IpDHN*, GenBank accession no.: KX426069) (data not published). In the current report, we focused mainly on the further functional characterization of *IpDHN* involved in salt and drought tolerance, as well as in ROS scavenging.

## Materials and Methods

### Plant Materials, Growth Conditions, and Stress Treatments

*Ipomoea pes-caprae* seeds were collected from the seaside area of Zhuhai City (22°16′25.37″N, 113°34′18.00″E), Guangdong Province, China. Different parts of *I. pes-caprae* were frozen with liquid nitrogen and stored in ultra-cold storage freezers (−80°C) until used for RNA isolation. For culturing the seedlings of *I. pes-caprae*, the seeds were sterilized with 70% ethanol followed by breaking of the seed coat with emery paper prior to placement on MS basal salts distributed on plates with sand and soil and placed outdoors from April to November in Guangzhou City. The seedlings were used for stress treatment assays to check the expression patterns of *IpDHN*. Subsequently, salt (300 mM NaCl), simulated drought or dehydration (300 mM mannitol), oxidative (0.1 mM methylviologen), cold (0°C) stresses, and ABA treatment (0.1 mM) were applied to the *I. pes-caprae* seedlings to detect the expression pattern of *IpDHN*. Experiments were performed following a completely randomized design with three replications and were repeated three times.

*Arabidopsis thaliana* (ecotype: Col-0) plants used for transgenic over-expression assay were grown on solid MS medium for approximately 10 days, and then transferred into soil. All plants were incubated in a growth green house with constant temperature (22°C) and relative humidity (70%) under a photoperiod of 16 h light/8 h darkness.

### Isolation of Full-Length *IpDHN* CDNA

A full-length cDNA library of *I. pes-caprae* was constructed and screened with a yeast salt-sensitive mutant (AXT3) (Zhou et al., 2015) complementary approach (data not published). Then, a full-length cDNA encoding DHN (IpDHN) that could rescue the phenotype of AXT3 was selected, and further investigations were performed.

### Sequence Analysis of the *IpDHN*

The CDS of *IpDHN* cDNA sequence was translated with online ORFfinder translator tool^1^. The S- and K-segments of IpDHN were characterized using the ExPASy prosite server^2^. The 3D prediction of IpDHN was also conducted with the online program^3^ PHYRE^2^ . MEGA6 was used to perform protein homology comparison and phylogenetic reconstruction by the neighbor-joining (NJ) method (Tamura et al., 2013). Bootstrap values were set with 1000 replicates to assess the relative support for each branch.

IpDHN protein (NCBI accession no.: AQZ36519.1) was aligned with known DHNs using ClustalW software^4^. The amino acid sequences of different DHNs were as follows: *Daucus carota* (DcDHN; NCBI accession no.: BAA82445.1), *Oryza sativa* (Os02g44870.1; NCBI accession no.: XP015627209.1), *Vitis vinifera* (VvDHN; NCBI accession no.: XP_002285919.1), *Solanum tuberosum* (StDHN; NCBI accession no.: AAB53203.1), *Capsicum annuum* (CaDHN; NCBI accession no.: NP_001311855.1), *Musa* ABB Group (MusaDHN-1; NCBI accession no.: AEI54683.1), *Coffea canephora* (CcDHN; NCBI accession no.: ABC68275.1), *Ipomoea nil* (InDHN; NCBI accession no.: XP_019187872.1), and 10 Arabidopsis dehydrins, including At1g76180.1, At1g20440.1, At1g20450.1, At4g38410.1, At3g50980.1, At2g21490.1 At4g39130.1, At3g50790.1, At1g54410.1, and At5g66400.1.

### Isolation of the Genomic Region and the Promoter of *IpDHN*

Two primers (IpDHNF and IpDHNR; **Supplementary Table S1**) were designed according to the full-length cDNA sequencing data of *IpDHN* to obtain the genomic region sequences of *IpDHN* with standard PCR amplification. The genomic DNA was extracted from seedlings of *I. pes-caprae* using the EasyPure Plant Genomic DNA Kit (TransGen Biotech, Beijing). PCR was performed for 5 min at 94°C, followed by 35 cycles of amplification (94°C for 30 s, 55°C for 40 s, and 68°C for 2 min) and 68°C for 10 min. Then, the purified PCR products were ligated into a pGEM T-vector (Promega, Shanghai) and were sequenced.

The 5′ flanking region upstream of the translation start codon (promoter sequence) of *IpDHN* was isolated from *I. pes-caprae* genomic DNA through genome walking using a Genome Walking Kit (Takara, Dalian) according to the manufacturer’s instructions. For nested PCR, the *IpDHN* gene-specific primers, IpDHNSP1, IpDHNSP2, and IpDHNSP3 (**Supplementary Table S1**), and the adaptor primer AP1 were used. The primers IpDHNSP1, IpDHNSP2, and IpDHNSP3 were designed according to the genomic sequence of *IpDHN* (including the intron). The *IpDHN* promoter was amplified via PCR and cloned into the pGEM-T Easy vector (Promega, Shanghai) for sequencing. The putative *cis*-acting elements of the *IpDHN* promoter were analyzed using the online tool PlantCARE^5^ (Lescot et al., 2002).

### Bacterial Overexpression and Salt, Osmotic, Dehydration, and H_2_O_2_ Tolerance Assays in *E. coli*

To further confirm the biological and biochemical functions of IpDHN, the exact ORF (open reading frame) of *IpDHN* cDNA was inserted into expression vector pGEX 6p-1 (GE Healthcare, Sweden) to generate the GST-tag recombinant plasmid IpDHN-pGEX 6p-1. The GST-IpDHN sequence was PCR amplified using the primers (IpDHNEPF and IpDHNEPR) listed in **Supplementary Table S1**. The PCR fragments were subsequently inserted into the *Bam*HI site of pGEX 6p-1, following the GST-tag with the in-fusion technique (BD In-Fusion PCR cloning Kit, Takara Bio USA), yielding the recombinant plasmid IpDHN-pGEX 6p-1. The recombinant plasmid and pGEX 6p-1 (as a negative control) were then transformed into *E. coli* BL21 (DE3). Then, two single colonies (containing IpDHN-pGEX 6p-1 or pGEX 6p-1 vectors) were inoculated in liquid LB medium and allowed to grow overnight at 37°C under constant shaking at 200 rpm. Inoculum (1%) from the culture grown overnight was added to fresh liquid LB medium (100 mL) containing 100 μg/mL of ampicillin and allowed to grow at 37°C and 180 rpm. Expressions were induced at an OD_600_ of 0.5 by 0.2 mM isopropyl β-D-thiogalactopyranoside (IPTG), and the *E. coli* cells were allowed to grow at 30°C for 2–6 h with constant shaking at 180 rpm. The induced bacteria were harvested and protein profiles were examined by 12% SDS PAGE.

A spot assay was carried out to confirm the tolerance of recombinant *E. coli*, with at least three replicates per sample. To assess salt, H_2_O_2_, and osmotic stresses, cell cultures of *E. coli* containing pGEX 6p-1/IpDHN-pGEX 6p-1 were adjusted to an OD_600_ value to 1.0 and then diluted gradiently (to 1:10, 1:100, and 1:1000). Two microliters of each sample was spotted onto the LB plates containing 0.2 mM IPTG and the stress treatment (5% NaCl, 6% NaCl, 2 M sorbitol, 5 mM H_2_O_2_, or 8 mM H_2_O_2_). For the drought-endurance test, 1 mL of OD-adjusted cell cultures was centrifuged, and the supernatant was discarded; the bacterial precipitate in tubes was immediately placed in a 40°C drying oven for 4 h. Then, the samples were added to 100 μL liquid LB medium and thawed at 37°C for 1 h to recover. Subsequently, the samples were diluted and spread out on LB plates containing 0.2 mM IPTG. The plates were placed into incubator at 37°C for 14 h. The bacterial colonies were counted with colony-forming units (cfu).

To determine the growth curve in liquid culture medium, 1 mL inoculum (OD_600_ = 1.0) was added to 10 mL LB medium (plus 0.2 mM IPTG) containing salt (3% or 4% NaCl), sorbitol (0.8 M or 1 M) or H_2_O_2_ (0.7 mM or 0.9 mM), and incubated at 37°C with shaking at 180 rpm. The aliquots were collected from each treatment sample every 2 h for a period of 12 h, and optical density (OD_600_) was recorded. Abiotic stress (salt, osmotic, and oxidative stress) tolerances were evaluated according to the *E. coli* growth status with respect to control cultures (bacterial cells and vector controls).

### Subcellular Localization Analysis

The CDS sequence (without the termination codon) of the *IpDHN* cDNA was amplified by PCR using the primer pair IpDHNGF and IpDHNGR (**Supplementary Table S1**). The PCR fragment was subcloned into the *Bam*HI site of the pUC/GFP vector to generate an IpDHN-GFP in-frame fusion protein under the CaMV 35S promoter, following the in-fusion technique (BD In-Fusion PCR Cloning Kit, Takara Bio USA), generating the recombinant plasmid IpDHN-pUC/GFP. After sequencing confirmation, the fusion construct or empty vector (as a control) and the NLS-mCherry vector were individually co-transfected into protoplasts (3 × 10^4^ protoplasts) using a PEG-calcium solution (0.4 g mL^−1^ PEG 4000, 0.2 M mannitol, 0.1 M CaCl_2_). After washing and resuspension in a W5 solution (154 mM NaCl, 125 mM CaCl_2_, 5 mM KCl, 5 mM glucose, 2 mM MES), mesophyll protoplasts were incubated under white light for 12–18 h. Fluorescence was visualized using a confocal laser scanning microscope (LSM 510 META, Zeiss, Germany).

### Quantitative RT-PCR Analysis

For detection of the expression of *IpDHN* in *I. pes-caprae* plants, total RNA was isolated from different tissues of *I. pes-caprae* with HiPure Plant RNA Kits (Magen, Guangzhou), and cDNA was synthesized from total RNA using TransScript One-Step gDNA Removal and cDNA Synthesis SuperMix (TransGen Biotech, Beijing) with Oligo (dT)_15_ primers according to the manufacturer’s instructions. QRT-PCR was conducted using a LightCycler^®^ 480 Gene Scanning system (Roche, Switzerland) and TransStart Tip Green qPCR SuperMix (TransGen Biotech, Beijing, China). The expression levels of *IpDHN* in various organs of seedlings and adult *I. pes-caprae* plants were detected, including the seedling root, seedling leaf, bud, mature root, vine, mature leaf, flower bud, petal, and young seeds 7 days after pollination (DAP). The *I. pes-caprae* seedling samples (roots, vines, and leaves), treated with salt, simulated drought or dehydration, oxidative and frost stresses and ABA, were also checked to examine the expression changes of *IpDHN*. Each tissue sample was collected from three dependent adjacent plants in the same location. All gene expression data obtained via quantitative RT-PCR were normalized to the expression of *IpUBQ* (GenBank Accession No.: MF502417). The primers used for quantitative RT-PCR are listed in **Supplementary Table S1**.

For detection of the expressions of antioxidant system-related genes (*CAT1*, *FSD1*, *CSD1*, and *APX2*) and abiotic stress-related genes (*NCED3*, *HAI2*, *RD29A*, *RD29B*, *HVA22D*, *ANAC19*, *RD22*, and *RD26*) in Arabidopsis (WT or transgenic plants), total RNA was isolated from rosette leaves at different time points (with or without treatments), and cDNA synthesis was performed following the above procedure. The reference gene for quantitative RT-PCR in Arabidopsis was *ACT2* (At3g18780). The primers used for quantitative RT-PCR are listed in **Supplementary Table S1**.

### DNA Constructs and Plant Transformation

To generate the recombinant vector for the overexpression assay in transgenic Arabidopsis, the full-length cDNA of *IpDHN* was PCR amplified using the primer pair IpDHNOXF and IpDHNOXR (**Supplementary Table S1**) to obtain the CDS region of *IpDHN*. The PCR product was cloned into the *Bam*HI site of the pBIm plasmid (Yang et al., 2005) to generate IpDHN-pBIm, with an expression cassette under the control of the CaMV 35S promoter.

After sequencing confirmation, the construct was transferred into *Agrobacterium tumefaciens* strain GV3101, the positive clone was picked out and cultured. The T-DNA region containing *IpDHN* and *NPTII* expression cassette was transformed into Arabidopsis with the floral dip method. Seeds of the T1 and T2 generations were germinated on MS agar medium containing 50 mg/L kanamycin to finally obtain homozygous lines. Positive transgenic plants were selected according to the segregation ratio (resistant:sensitive = 3:1) and were confirmed by genomic PCR with the primer pair IpDHNOXF/IpDHNOXR.

### Abiotic Stress Tolerance Assays in Transgenic Arabidopsis

The seed germination rate of *IpDHN* transgenic Arabidopsis was detected under NaCl (100, 125, 150, 175, and 400 mM) and mannitol (200, 300, and 400 mM) challenges to detect the positive effect of *IpDHN* overexpression on the improvement in the salt and osmotic tolerance of transgenic Arabidopsis seed germination. In addition, the root length was also measured to evaluate the influence of *IpDHN* overexpression on the seedlings of transgenic Arabidopsis under abiotic stresses. WT Arabidopsis was used as a control.

Salt and drought tolerance assays were also assesses with transgenic Arabidopsis adult plants. Both WT and transgenic seeds were synchronously germinated on MS medium plates. Ten-day-old seedlings were transplanted into sieve-like square pots filled with soil mixture and were well watered. Thirty plants of each genotype were planted in a growth pots and trays placed in greenhouse as described above without watering for 10 days. Subsequently, the plants were subjected to the following assays. For drought-tolerance assays, WT and transgenic plants (*IpDHN OX1,* and *IpDHN OX2*) were maintained under a continuous drought for 9 days and were re-watered for 7 days. For salt-tolerance assays, plants of each genotype were planted in sieve-like pots and were well watered as described for the drought-tolerance treatment. Water was withheld for 10 days before irrigation with NaCl solution (150 mM and 200 mM) from the bottom of the trays. When the soil was completely saturated with salt water, the NaCl solution was removed and the plants were cultured normally. The plants grew in salt-dosed soils for 10 days and were re-watered with fresh water for 7 days.

### Measurement of Physiological Parameters

Rosette leaves were gathered from 3-week-old seedlings growing in soil in a greenhouse at room temperature, with or without treatments, including 200 mM NaCl for 24 h or 300 mM mannitol for 24 h. Subsequently, several plant physiological parameters were detected and calculated, including RWC (relative water content), IL (ion leakage), the proline (Pro) content, the MDA content, CAT activity, and SOD activity. Experiments were performed following a completely randomized design with three replications and were repeated three times.

The RWC was calculated as described previously (Hu et al., 2013). Fresh weight (FW) of Arabidopsis rosette leaves were recorded followed by soaking the leaves for 4 h in distilled water at room temperature with constant light. After this treatment the turgid weight (TW) was recorded. The leaves were then dried for 24 h at 80°C to obtain the total dry weight (DW). RWC was calculated from the equation: RWC (%) = [(FW–DW)/(TW–DW)] ^∗^ 100.

Ion leakage was measured with a DDS-307A conductivity meter (Shanghai Jingke, China), according to Hu et al. (2013) with slight modifications. Arabidopsis rosette leaves were cut into strips (1 mm) and incubated in 10 mL double distilled water at room temperature, shaking at 150 rpm for 2 h. Initial conductivity (C1) was measured with a conductivity meter followed by boiling the samples for 30 min to measure complete IL. The leaves were then cooled to room temperature to measure the electrolyte conductivity (C2). The electrolyte conductivity of double distilled water was also measured (C0). IL was calculated according to the equation: IL (%) = (C1–C0)/(C2–C0) ^∗^ 100.

The free proline (Pro) content, malondialdehyde (MDA) content, and SOD and CAT activities were determined using Proline, MDA, SOD, and Catalase Assay Kits, respectively, according to the manufacturer’s instructions (Nanjing Jiancheng Bioengineering Institute, China).

Rosette leaves were collected from 2-week-old seedlings growing in soil; the leaf stalks were immersed in 150/200 mM NaCl or 300 mM mannitol solutions for 3 h. *In situ* detection of H_2_O_2_ and O_2_^−^ was determined using vacuum-infiltration with 0.2% nitro-blue tetrazolium (NBT) or 1 mg/mL 3.3′-diaminobenzidine (DAB) solution, respectively, for 12 h followed by clearing in 75% ethanol, as previously described (Cao et al., 2017).

### Statistical Analysis

All of the experiments in this study were repeated three times in independent experiments, and the data shown are the mean ± SD. In this research, statistical analyses were performed using the statistical tools (Student’s *t*-test) of Microsoft Excel software.

## Results

### *IpDHN* Encodes an SK_3_ Type Dehydrin

*IpDHN* and other candidate salt stress-related genes were identified from a cDNA full-length library, generated from total RNA of *I. pes-caprae* seedlings, based on the FOX gene hunting system (unpublished data), screened with a salt-sensitive yeast mutant strain, AXT3 (Zhou et al., 2015). The full-length *IpDHN* cDNA was inserted into the pYES-DEST-52 expression vector under the control of a galactose-induced promoter, P_GAL_. Our previous research indicated that the induced expression of *IpDHN* in yeast could partly rescue the salt-sensitive phenotype of the AXT3 yeast mutant and also improved the salt tolerance of the WT yeast W303. We also found that *IpDHN* could elevate the tolerance of yeast strains (*yap1*Δ and *skn7*Δ) to oxidative stress (**Supplementary Figure S1**).

The full-length *IpDHN* cDNA is 983 bp, including an 85-bp 5′ -untranslated region (UTR), a 244-bp 3′ -UTR region and a 654-bp ORF (**Supplementary Figure S2A**). Analysis of the protein sequence using the ExPASy ProtParam calculated the isoelectric point to be 5.34, with a molecular mass of 24.162 kD and an overall hydropathicity average (GRAVY) of −1.364, indicating that IpDHN is strongly hydrophilic. The amino acid composition of the IpDHN protein is shown in **Supplementary Table S2**.

**FIGURE 1:**
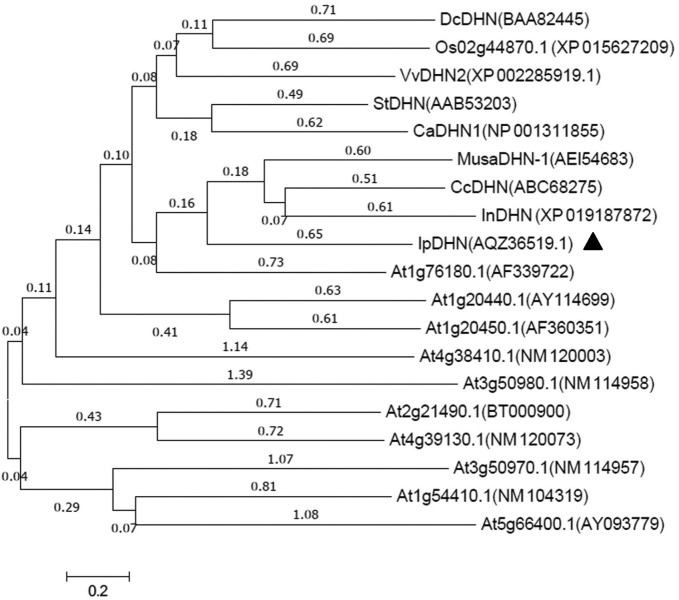
Phylogenetic relationships between the IpDHN protein and dehydrins from other plant species. The molecular phylogeny was constructed from a complete protein sequence alignment of DHNs by the neighbor-joining method with ClustalW. The numbers beside the branches indicate bootstrap values. The symbol “
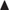
” shows the position of IpDHN in the phylogenetic tree.

SmartBLAST analysis with the amino acid sequence of IpDHN showed that it is highly homologous to DHN InDHN from *Ipomoea nil*, CcDHN from *Coffea canephora*, CaDHN1 from *Capsicum annuum*, and StDHN from *Solanum tuberosum* (**Supplementary Figure S2B**). Further analysis showed that the IpDHN protein contained an S segment and three K segments (**Supplementary Figure S2B**). The IpDHN protein was rich in hydrophilic amino acids such as Glu (22.58%), Lys (18.89%), Ser (6.45%), and His (4.61%), but lacked the hydrophobic amino acid Trp (0) (**Supplementary Table S2**). In addition, *in silico* prediction with the Protein Fold Recognition Server tool (PHYRE^2^) suggested that more than 80% of the amino acid residues of IpDHN were disordered, indicating that IpDHN is an IDP. The interactive 3D view in JSmol (PHYRE^2^) showed that IpDHN was highly disordered (**Supplementary Figure S3**). A phylogenetic tree was constructed to evaluate the molecular evolutionary relationships between IpDHN and other plant DHN proteins. The results showed that the IpDHN protein can be grouped as the SKn type (**Figure 1**).

### The Structure of the *IpDHN* Genomic Sequence of the Coding and Promoter Regions

**FIGURE 2:**
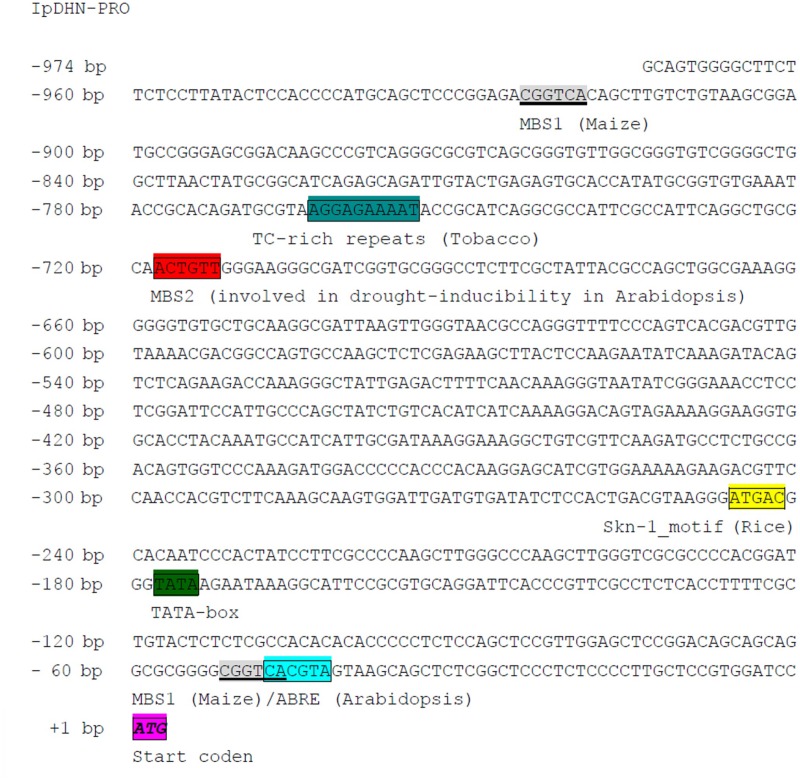
Nucleotide sequence of the *IpDHN* promoter (974 bp). Nucleotides are numbered on the left. The putative translation start sites, TATA box, and other important *cis*-regulatory elements are boxed and labeled.

A comparison of genomic (**Supplementary Figure S2C**) and cDNA (**Supplementary Figure S2B**) sequences showed that the *IpDHN* contained a single intron of 264 bp. We also isolated a 974 bp IpDHN promoter region. Analysis of the *IpDHN* promoter using PlantCARE^6^ indicated that numerous potential *cis*-acting elements that respond to environmental signals were present (**Figure 2**), and these elements are classified in **Supplementary Table S3**. A core promoter element, i.e., a TATA-box sequence, was located 178 bp upstream of the translational start site (TSS). We also identified an ABA-responsive element (ABRE, TACGTG sequence, 48 bp upstream of TSS) and three Myb binding sites (MBS1 and MBS2, CGGTCA/CAACTG sequences, 52, 718, and 925 bp upstream of TSS), which might be involved in drought induction and other abiotic stresses. A typical *cis*-acting element Skn-1-motif (GTCAT), proven to be capable of activating gene expression in the endosperm, was found 246 bp upstream of TSS, which further implicated IpDHN in belonging to seed storage proteins, matching the characteristics of LEA proteins. Furthermore, a TC-rich repeat regulatory element (ATTTTCTCCA), with homology to that identified in defense and stress-responsive genes, was detected 767 bp upstream of TSS (**Figure 2**). The other *cis*-elements in the *IpDHN* promoter region are listed in **Supplementary Table S3**.

### Salt, Dehydration and H_2_O_2_ Tolerance Assays of *E. coli*

**FIGURE 3:**
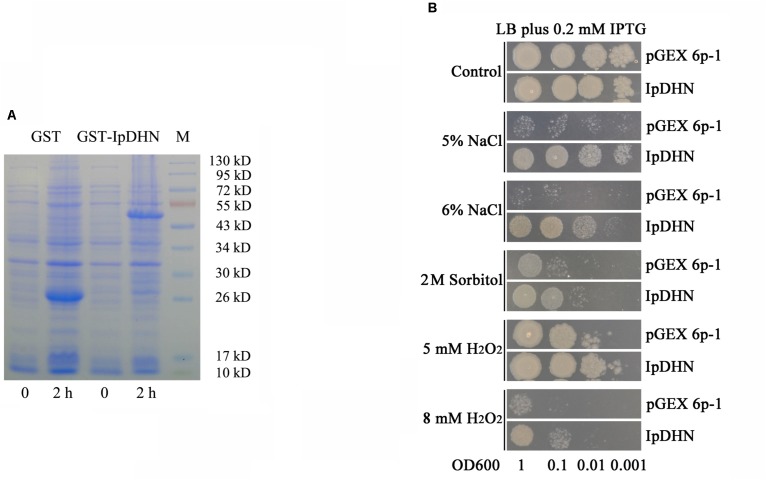
Functional analysis of IpDHN induced-expression for salt, drought, and H_2_O_2_ tolerance in *Escherichia coli*. **(A)** Induced expression of the GST-IpDHN (IpDHN- pGEX 6p-1) fusion protein and single GST (pGEX 6p-1 empty vector) protein in *E. coli*. 0 and 2 h: the IPTG induction time. **(B)** The growth performance of *E. coli* BL21 (pGEX 6p-1, upper)/(IpDHN-pGEX 6p-1) on LB plates containing stress factors. Control (top): LB medium; 5% NaCl: LB medium containing 5% NaCl; 6% NaCl: LB medium containing 6% NaCl; 2 M Sorbitol: LB medium containing 2 M sorbitol; 5 mM H_2_O_2_: LB medium containing 5 mM H_2_O_2_; 8 mM H_2_O_2_: LB medium containing 8 mM H_2_O_2_. The cell cultures were adjusted to OD_600_ to 1 and were then diluted serially (1:10, 1:100, and 1:1000, respectively). Two microliters of each sample was spotted onto the LB plates containing 0.2 mM IPTG.

The coding region of *IpDHN* cDNA was amplified and cloned into the pGEX 6p-1 vector (*Bam*HI site). After sequence confirmation, the recombinant plasmid IpDHN-pGEX 6p-1 and empty vector pGEX 6p-1 (as a negative control) were expressed in *E. coli* BL21 (DE3) cells. The GST-IpDHN and GST proteins were analyzed by SDS-PAGE (12%). As shown in **Figure 3A**, obvious expression of recombinant IpDHN (fused with GST) and the GST protein was observed after 2 h, compared with non-induced cells. The fusion protein (GST-IpDHN) presented a induced band of approximately 50 kDa, which is accordant with the expected size of GST-IpDHN, which was undetectable in non-induced *E. coli* cells (**Figure 3A**), indicating that the GST-IpDHN fusion protein was correctly expressed in recombinant *E. coli*. The difference in the molecular weight between the fusion and GST proteins confirmed the predicted 24-kDa molecular weight of the IpDHN protein.

The bacteria spot and liquid culture assays were performed with IPTG-induced *E. coli* cells to confirm the positive effect of IpDHN’s over-accumulation on salt, osmotic, and oxidative stresses tolerance. As can be observed in **Figure 3B**, obvious differences in *E. coli* growth status were observed for the spot assay, where the numbers of colonies were much higher in several stress treatments for recombinant *E. coli* cells with induced GST-IpDHN compared to *E. coli* cells with the empty vector (GST) control. The *E. coli* liquid culture growth curve assay under stress conditions indicated that the growth of *E. coli* cells accumulating the GST-IpDHN fusion protein increased significantly and rapidly compared to *E. coli* cells expressing the GST protein only (**Supplementary Figure S4A**).

The tolerance of *E. coli* with the empty vector (GST) control and GST-IpDHN subjected to drought stress was also determined by spreading dilutions of cultures on LB plates containing 0.2 mM IPTG, after drying the bacterial precipitate at 40°C for 4 h and recovering the precipitates in 100 μL liquid LB medium for 1 h. The Colony-Forming Units (cfu) counting assay shows a significant difference in the number of living *E. coli* cells before and after desiccation stress (**Supplementary Figure S4B**). Before the drying process, the control (GST) and GST-IpDHN expressing *E. coli* cells showed very similar colony numbers (CFU, expressed in ×10^6^ units), but after desiccation, although only a very small fraction of cells survived, the number of colonies expressing GST-IpDHN was six times higher than control cells. Our result here indicates that IpDHN’s induction in *E. coli* cells improved their survival capacity after desiccation.

### Subcellular Localization of IpDHN

**FIGURE 4:**
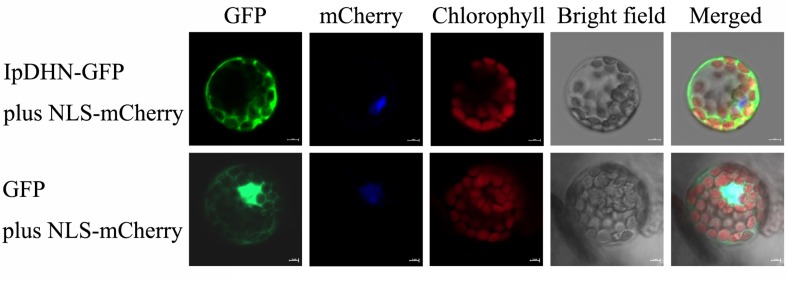
Subcellular localization of IpDHN. Arabidopsis protoplasts expressing the 35S:IpDHN-GFP fusion protein **(upper)** and 35S:GFP **(lower)** observed under a laser scanning confocal microscope. The blue color indicates the nucleus using mCherry as the nuclear marker. The red color indicated autofluorescence emitted by chloroplasts. Bar represents 5 μm.

The subcellular localization of IpDHN was determined by a GFP-fusion protein transient expression assay in Arabidopsis mesophyll protoplasts. The results showed that IpDHN-GFP primarily distributed in the entire cytoplasm of Arabidopsis mesophyll protoplasts (**Figure 4**, upper row), with a similar localization pattern for the control GFP (**Figure 4**, lower row). However, the IpDHN-GFP was clearly missing in the nucleus, but the control GFP exhibited strong signals in the nucleus, which were detected throughout the cell (**Figure 4**). Based on these *in vivo* targeting results, we concluded that IpDHN is predominantly localized to the cytoplasm but might not undergo nuclear distribution.

### Quantitative RT-PCR Analysis of IpDHN Under Abiotic Stresses and ABA Treatment

**FIGURE 5:**
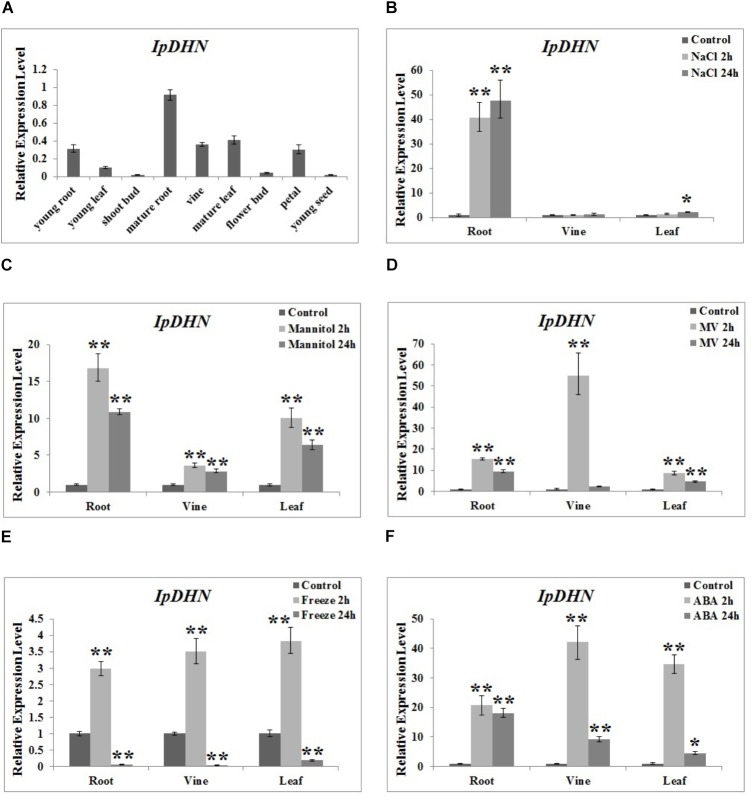
Expression profiles of the *IpDHN* gene among *Ipomoea pes-caprae* tissues. **(A)** Differential expression of *IpDHN* in various tissues (young root, young leaf, shoot bud, mature root, vine, mature leaf, flower bud, petal, young seed). **(B–F)** Time-course expression patterns of *IpDHN* in response to different abiotic stresses: NaCl **(B)**, Mannitol **(C)**, MV **(D)**, freeze **(E)**, and ABA treatment **(F)**. Error bars indicate the SD based on three replicates. Asterisks indicate significant differences from the control (Student’s *t*-test *P*-values, ^∗^*p* < 0.05, ^∗∗^*p* < 0.01).

To investigate the expression pattern of *IpDHN* in *I. pes-caprae*, quantitative RT-PCR was performed with total RNAs extracted from various tissues. Our results demonstrated that *IpDHN* was expressed constitutively in most *I. pes-caprae* tissues (**Figure 5A**). The highest level of the *IpDHN* transcript was detected in the mature root, vine and leaf; the young root and flower petal also showed a high expression of *IpDHN*, while *IpDHN* was weakly expressed in the tissues/cells that were rapidly growing, dividing and metabolizing, such as the young leaf, shoot bud, flower bud, and young seed. The expression pattern of *IpDHN* indicated that the biological role of *IpDHN* was more involved in abiotic stress response as opposed to cellular growth and development.

To determine the expression of *IpDHN* under stress, the transcript of *IpDHN* in the root, vine, and leaf of *I. pes-caprae* seedlings was analyzed by quantitative RT-PCR. Under 300 mM NaCl salt stress, the transcript level of *IpDHN* showed the greatest increase to more than 40-fold in the root and slight increases in the vine and leaf tissues (**Figure 5B**). Under dehydration stress (300 mM mannitol, simulating drought stress), the whole *I. pes-caprae* seedling showed an increased expression pattern of *IpDHN*, and in the root, the induced level reached almost 20-fold (**Figure 5C**). Low temperature (0°C) could also rapidly and slightly induce the expression of *IpDHN*, with a peak at 2 h (**Figure 5E**). We also checked the expression change of *IpDHN* under methyl viologen (oxidative stress) and ABA treatment (**Figures 5D,F**). Our results showed that both methyl viologen and ABA could greatly and rapidly increase the transcript level of *IpDHN* (**Figure 5**).

### Overexpression of *IpDHN1* in Arabidopsis Enhances Tolerance to Salt and Drought

**FIGURE 6:**
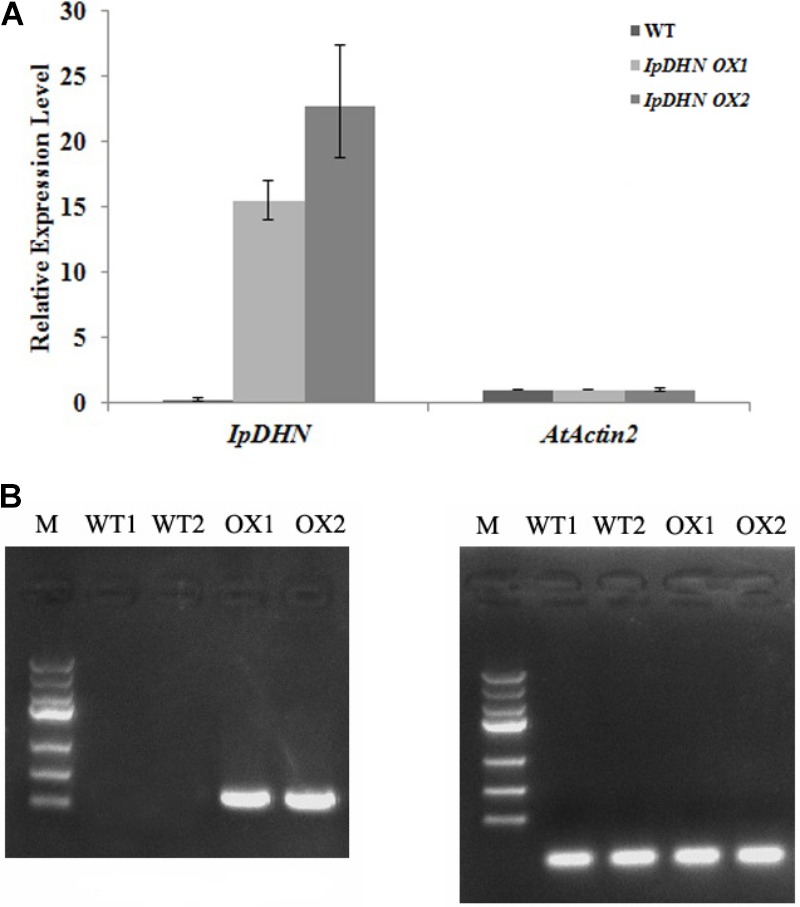
The overexpression analyses of *IpDHN* in transgenic Arabidopsis lines (*IpDHN OX1* and *IpDHN OX2*). **(A)** Quantitative RT-PCR analysis of *IpDHN* in transgenic Arabidopsis lines and WT Arabidopsis (WT). *AtActin2* was used as an internal control. Error bars indicate the SD based on three replicates. **(B)** RT-PCR analysis of *IpDHN* in transgenic Arabidopsis lines and two WT lines. **(C)** RT-PCR analysis of *AtActin2* in transgenic Arabidopsis lines and two WT lines as a control.

To further determine its biological function, *IpDHN* was over-expressed in Arabidopsis. Two homozygous T3 lines (*IpDHN OX1* and *IpDHN OX2*) were selected for *IpDHN,* and quantitative RT-PCR was performed. The results showed that both *IpDHN* overexpression lines were highly expressed in the transgenic Arabidopsis (**Figure 6A**). In addition, the RT-PCR results also showed that the expression of *IpDHN* (*IpDHN OX1* and *IpDHN OX2*) was higher than that of the reference gene *AtActin2* (At3g18780) in two transgenic lines (**Figure 6**).

**FIGURE 7:**
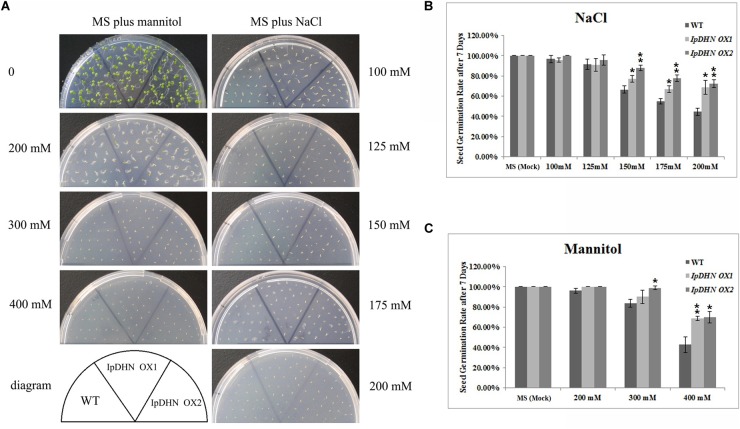
Osmotic and salt stress analyses of transgenic plants with *IpDHN* with respect to the seed germination rate. **(A)** Photographs of transgenic lines (*IpDHN OX1* and *IpDHN OX2*) and WT seeds germinated on MS medium or MS medium with mannitol (left, 200, 300, and 400 mM) or NaCl (right, 100, 125, 150, 175, and 200 mM) for 7 days. Seed germination rates were calculated for the WT and transgenic lines under NaCl **(B)** and mannitol **(C)** stress after 7 days. Error bars indicate the SD based on three replicates. Asterisks indicate significant differences from the WT (control, Student’s *t*-test *P*-values, ^∗^*p* < 0.05, ^∗∗^*p* < 0.01).

To detect whether the over-accumulation of *IpDHN* in transgenic Arabidopsis could affect osmotic stress tolerance at seedling emergence stage, the seed germination rates of *IpDHN OX1*, *IpDHN OX2* and the WT were monitored under different concentrations of NaCl or mannitol challenges. As shown in **Figure 7**, under non-stress conditions or a low level of osmotic stress (100 mM NaCl, 100 or 125 mM mannitol), no significant differences in the seed germination rate were observed between the WT and transgenic plants; however, when grown on MS medium supplemented with a higher concentration of NaCl (150, 175, and 200 mM) or mannitol (300 and 400 mM), the transgenic seeds (*IpDHN OX1* and *IpDHN OX2*) showed significantly higher germination rates than those of the WT. At a low level of osmotic stress (100 mM NaCl, 100 or 125 mM mannitol), the transgenic seedlings showed significantly larger cotyledons than those of the WT, even with similar seed germination rates (**Figure 7**). The obvious effect of *IpDHN* on improving osmotic stress during seed germination was also observed when the seeds were challenged continuously with 150 mM NaCl or 200 mM or 300 mM mannitol for 3 weeks (**Supplementary Figure S5**).

**FIGURE 8:**
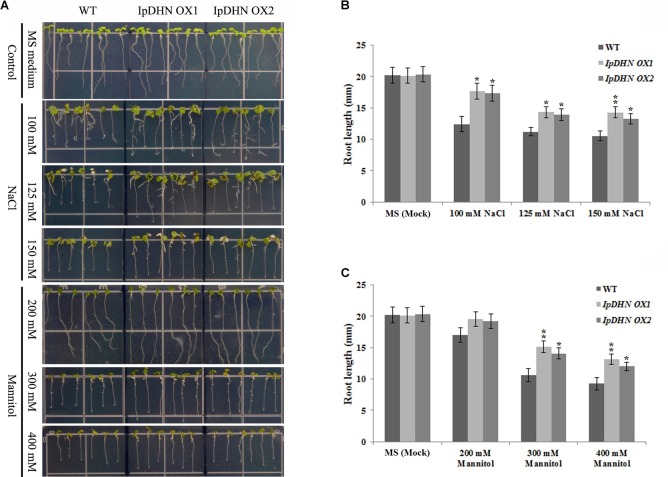
Osmotic and salt stresses analyses of transgenic plants with *IpDHN* with respect to seedling root length. Four-day-old seedlings were transplanted to MS medium containing NaCl or mannitol and were then grown for 7 days before measuring the root length. **(A)** Photographs of transgenic lines (*IpDHN OX1* and *IpDHN OX2*) and WT seedlings on MS medium or MS medium with NaCl (right, 100, 125, and 150 mM) or mannitol (200, 300, and 400 mM). **(B,C)** Seedling root length of WT and transgenic lines under NaCl **(B)** and mannitol **(C)** stress after 7 days. Error bars indicate the SD based on three replicates. Asterisks indicate significant differences from the WT (control, Student’s *t*-test *P*-values, ^∗^*p* < 0.05, ^∗∗^*p* < 0.01).

To further test osmotic tolerance, the root length of Arabidopsis seedlings was also measured to assess the tolerance of plant growth. Four-day-old seedlings were transferred onto MS/agar plates containing NaCl or mannitol and were further cultivated vertically for 7 days after which the root lengths were measured. As we can see from **Figure 8**, there are no significant differences in root length were observed between the WT and transgenic lines when grown on medium without NaCl or mannitol. Correspondingly, for the Correspondingly, for the challenges of medium containing 125 mM NaCl or 200 mM mannitol, the root lengths of both sets of transgenic plants were significantly longer than those of the WT plants, suggesting that the overexpression of *IpDHN* enhances salt and drought/dehydration tolerance at the seedling stage (**Figure 8**).

**FIGURE 9:**
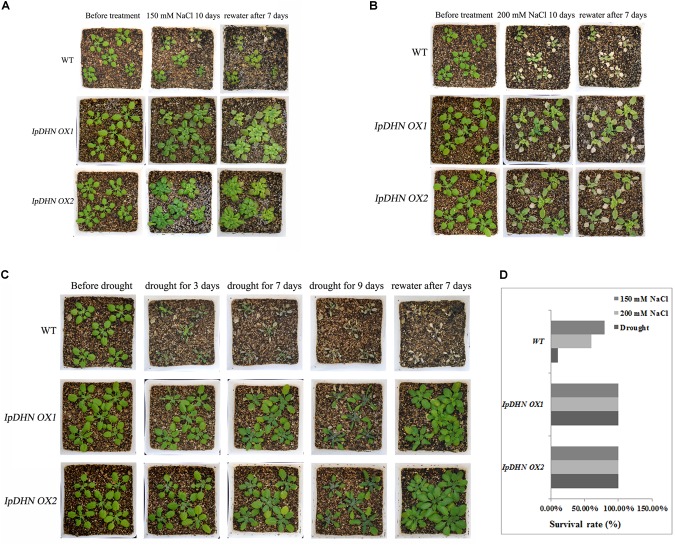
Photographs and survival rate of the transgenic lines (*IpDHN OX1* and *IpDHN OX2*) and WT plants grown in pots under normal and salt/drought conditions. **(A)** The effects of 150 mM NaCl on transgenic lines and WT. **(B)** The effects of 200 mM NaCl on transgenic lines and WT. **(C)** The effects of water withholding on transgenic lines and WT. **(D)** The statistics for the survival rate of transgenic lines and WT Arabidopsis after salt/drought stresses. Thirty plants from the WT and from the two transgenic lines (*IpDHN OX1* and *IpDHN OX2*) were treated with various concentrations of NaCl or drought.

We also conducted salt and drought stress assays on adult Arabidopsis plants to further characterize the phenotypes of *IpDHN* overexpression in Arabidopsis. In general, we characterized the phenotypes of the *IpDHN* overexpression lines through adult-plant stress assay in pots (**Figure 9**). The seedlings of these lines were transferred to well-watered soil in pots, and watering was withheld for approximately 10 days to gradually reduce the water content of soil. Two levels of high salinity, 150 and 200 mM NaCl infiltrated soil, were taken in this assay. Under normal conditions, no differences in the growth of the transgenic lines and the WT controls were observed. While after challenges of salinity stress, almost all of the WT plants showed a severe reduction of growth (**Figures 9A,B**). We observed severe dehydration of the leaves, and the whole plant was wilted. By contrast, although some of the leaves of most of the DHN transgenic plants showed chlorosis, as a whole, they showed resistance to salinity stress and were still able to grow.

The *IpDHN* transgenic plants also showed improvement of drought tolerance. The adult Arabidopsis plants (including *IpDHN OX1*, *IpDHN OX2* and WT), from which watering was withheld for 10 days, were adapted for the drought-tolerance assay. Along with the gradual water loss, the majority of the WT plants exhibited obvious wilting due to severe stress while the survival rate of the transgenic lines (*IpDHN OX1* and *IpDHN OX2*) was apparently higher than that of the WT controls (**Figure 9C**). After the rewatering treatment, the *IpDHN OX* lines were largely restored, while the WT controls showed a lethal phenotype (**Figure 9C**).

The above results indicated that *IpDHN* increased plant tolerance to salinity and drought stress and significantly improved the survival rates of transgenic Arabidopsis under osmotic stress (**Figure 9D**). These results also indicated that the accumulation of IpDHN in transgenic Arabidopsis provides better protection for basic cellular activities *in vivo* under osmotic stress.

### Overexpression of *IpDHN* Increases RWC, Proline Content and Decreases IL and the MDA Content Under Osmotic Stresses

**FIGURE 10:**
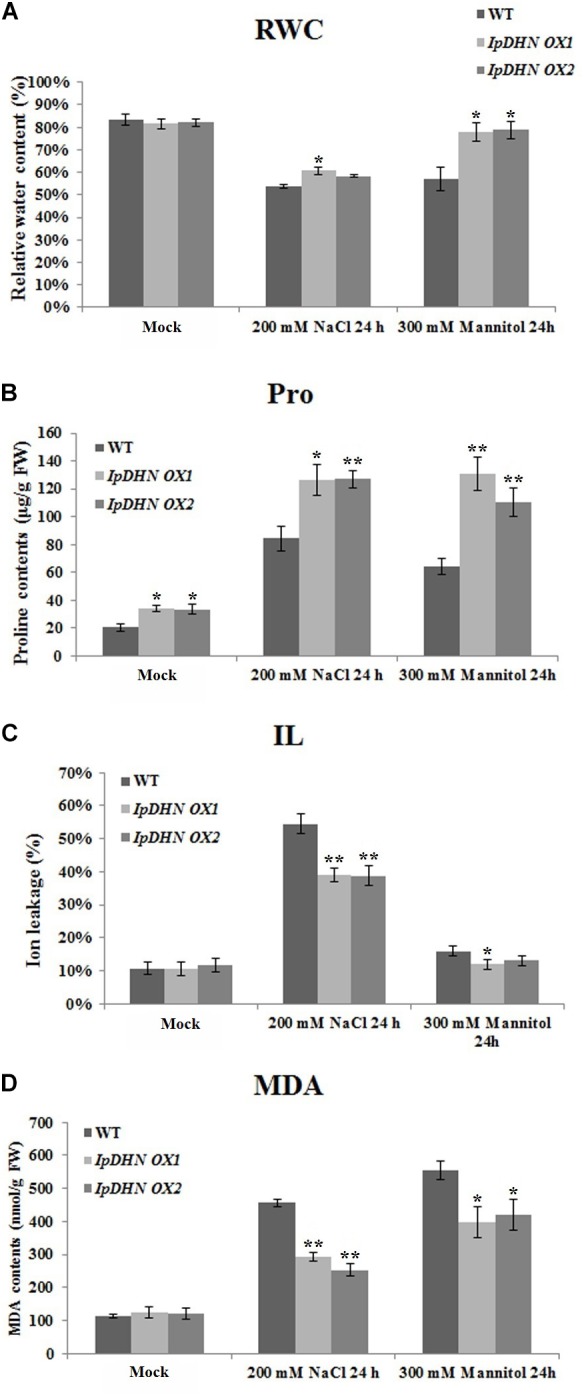
Changes in physiological parameters of *IpDHN* overexpressing Arabidopsis and WT seedlings (4 weeks) under 200 mM NaCl and 200 mM mannitol treatments for 24 h. **(A)** RWC; **(B)** free proline content; **(C)** IL; **(D)** MDA. All determinations were carried out on three biological replicates. Error bars indicate the SD based on three replicates. Asterisks indicate significant differences from the WT (control, Student’s *t*-test *P*-values, ^∗^*p* < 0.05, ^∗∗^*p* < 0.01).

To further clarify the possible physiological mechanisms involved in cellular protection mediated by IpDHN, several physiological indices, including RWC, IL, the proline (Pro) content, and MDA content, which are mainly related to cellular osmotic stress tolerance, were tested in WT and *IpDHN* transgenic Arabidopsis plants under salt and osmotic stress treatments. Compared to the WT Arabidopsis plants, the RWC and proline content was a little higher than those in transgenic lines when they were subjected to salt stress (200 mM NaCl, 1 day) or osmotic stress (300 mM mannitol, 1 day **Figures 10A,B**), which indicated that in transgenic lines, the cells had better water status and stress resistance than in the WT plants. IL is an important indicator of membrane injury and represents the integrity of cell biomembrane system. The IL value was higher in WT than in the transgenic lines, suggesting that the transgenic plants suffered less membrane damage than WT (**Figure 10C**). Accordingly, the MDA levels also displayed a pattern similar to IL, being lower in the transgenic lines relative to WT (**Figure 10D**), probably mediated by the cellular IpDHN’s accumulation. These physiological indices indicated that the transgenic lines were more resistant to salt and drought stresses, with an indication of the IpDHN protection roles for the cellular membrane system, even for cell vitality under osmotic stresses.

### *IpDHN* Transgenic Overexpressing Arabidopsis Resist Oxidative Damage by Increasing ROS Scavenger Accumulation

**FIGURE 11:**
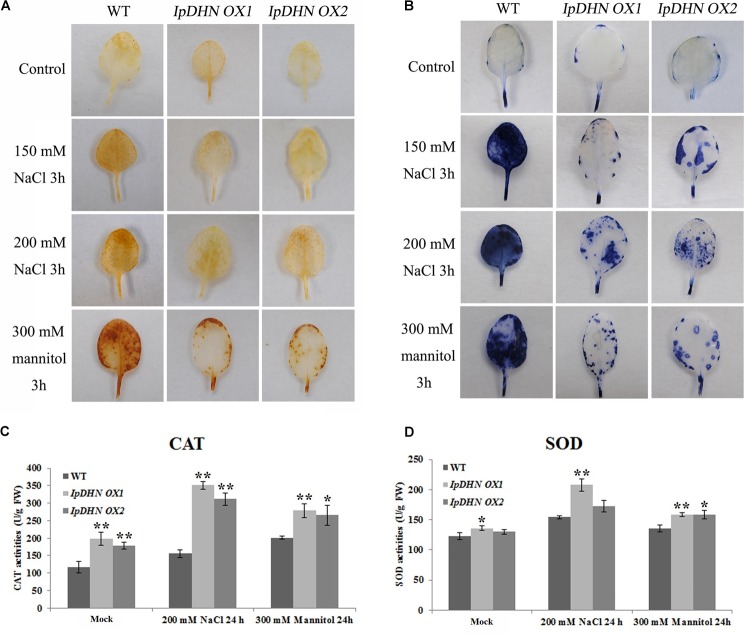
Oxidative stress analyses of transgenic lines and WT plants. Histochemical staining assays were used to detect H_2_O_2_ and O_2_^−^ by DAB **(A)** or NBT **(B)** staining, respectively. **(C)** Analysis of CAT activities in the WT and transgenic lines under normal conditions and osmotic stresses. **(D)** Analysis of SOD activities in the WT and transgenic lines under normal conditions and osmotic stresses. Error bars indicate the SD based on three replicates. Asterisks indicate significant differences from the WT (control, Student’s *t*-test *P*-values, ^∗^*p* < 0.05, ^∗∗^*p* < 0.01).

From **Figure 10**, we can see that the proline content (**Figure 10A**) and MDA content (**Figure 10C**) showed obvious differences between the WT and *IpDHN* overexpression plants, which means that the cells of the *IpDHN* transgenic plants showed stronger activity and better resistance to osmotic stresses. The proline and MDA contents are also important indices of cellular oxidative stress, as well as cell injury in response to stresses (Ben Rejeb et al., 2014; Yuan et al., 2017). Abiotic stresses, such as drought, cold, salt and heat, could cause increases in the levels of ROS in plant *in vivo*, which subsequently result in a reduction of plant growth and even loss of crop yield worldwide (You and Chan, 2015). When plant cells are subjected to stresses, proline can act as an osmolyte as well as a ROS scavenger (Ben Rejeb et al., 2014), whereas the accumulation of MDA is often the final product of membrane lipid peroxidation caused by ROS accumulation. In other words, the differences in the physiological indices between the WT and *IpDHN* transgenic plants are eventually reflected in the cellular ROS accumulation and scavenging caused by osmotic stresses. Hence, it was necessary to explore ROS accumulation in the *IpDHN* transgenic lines and WT. From **Figure 11**, we can see that the H_2_O_2_ and O_2_^−^ contents increased in both the WT and *IpDHN* transgenic lines after salt and mannitol (osmotic) stresses. The DAB (**Figure 11A**) and NBT (**Figure 11B**) staining assays indicated that the two transgenic line plants contained less H_2_O_2_ and O_2_^−^ than WT. Some enzymatic antioxidants, such as CAT and SOD, might play significant roles in ROS scavenging and may influence the cellular ROS balance. In our research, the activities of two significant antioxidant enzymes (SOD and CAT) were measured in the leaves of potted plants. Under normal growth conditions, the *IpDHN* transgenic lines and WT plants showed no apparent difference in SOD activity, while the CAT activity was slightly higher in the *IpDHN* transgenic lines than in the WT. However, the SOD and CAT activities were significantly higher in the *IpDHN* transgenic lines than in the WT plants (**Figures 11C,D**). These results suggested that overexpression of *IpDHN* reduced ROS accumulation, probably by the protection roles to basal metabolisms of IpDHN’s accumulation, and then by some means enhancing the cellular SOD and CAT activities under salt/dehydration stresses, which eventually can help to lessen the poisonous effect of ROS.

### Overexpression of *IpDHN* Induces Expression of Stress-Responsive Genes

**FIGURE 12:**
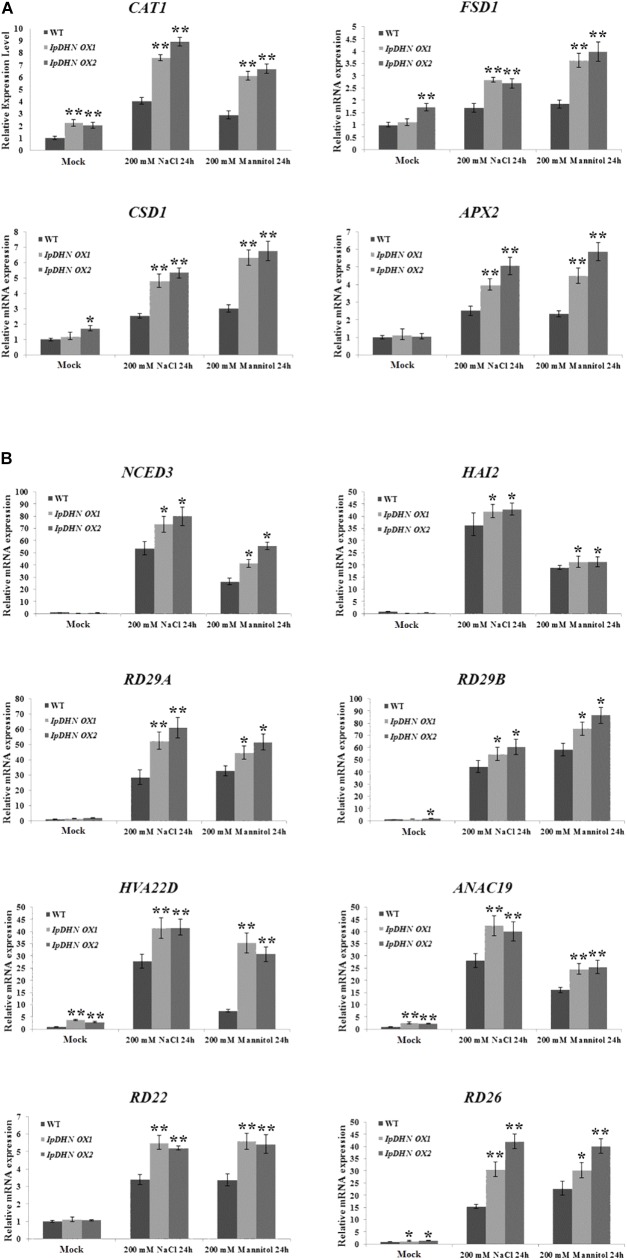
Analysis of expression levels of ROS-related and stress-responsive genes in the WT and the transgenic line by qRT-PCR under normal and osmotic conditions. **(A)** *CAT1*, *FSD1*, *CSD1*, and *APX2*; **(B)** *NCED3*, *HAI2*, *RD29A*, *RD29B*, *HVA22D*, *ANAC19*, *RD22*, and *RD26*. Error bars indicate the SD based on three replicates. Asterisks indicate significant differences from the WT (control, Student’s *t*-test *P*-values, ^∗^*p* < 0.05, ^∗∗^*p* < 0.01).

To explore the molecular mechanisms underlying the biological function of *IpDHN* in salt and drought resistance in plants, the expression levels of a number of antioxidative and water deficit-responsive genes in the *IpDHN* transgenic lines and WT plants subjected to 200 mM mannitol and 200 mM NaCl treatments were analyzed by quantitative RT-PCR (**Figure 12**). The four oxidative stress-responsive marker genes (*CAT1*, *FSD1*, *CSD1*, and *APX2*) examined in Arabidopsis showed significantly upregulated transcription in the WT and *IpDHN* transgenic plants under stress conditions (**Figure 12A**). Moreover, the other eight drought/dehydration or ABA responsive genes (*NCED3*, *HAI2*, *RD29A*, *RD29B*, *HVA22D*, *ANAC19*, *RD22*, and *RD26*) showed an upregulated expression pattern in *IpDHN* transgenic Arabidopsis compared with the WT plants. In addition, under osmotic stresses, these genes showed a greater increase of the induced expression pattern in *IpDHN* transgenic Arabidopsis compared with the WT plants (**Figure 12B**). This result suggested that IpDHN accumulation in plants might up-regulate the expression of these abiotic stress-related genes, and then these upstream regulatory genes can improve plant resistance to salt and drought stresses in some way.

## Discussion

In plants, DHNs function in various pathways, conferring water-deficit stress tolerance. Plant DHN cDNAs were first identified in barley and corn seedlings undergoing dehydration treatment, with induced expression pattern under ABA and salt treatment (Close et al., 1989). Subsequently, a series of plant DHNs have been reported. Plant DHNs that have been functionally characterized and identified are involved in responses to abiotic stresses, not only in the model plant Arabidopsis and crops (Hara, 2010; Eriksson et al., 2011; Hanin et al., 2011; Kosová et al., 2014), but also in some wild plants, especially those with tolerance to extremely adverse conditions, such as halophytes (Ayarpadikannan et al., 2012; Yamamoto et al., 2015), drought-enduring plants (Yang et al., 2014; Chiappetta et al., 2015), resurrection plants (Giarola et al., 2015), and hardy plants (Ochoa-Alfaro et al., 2012; Bao et al., 2017; Guo et al., 2017). In this study, a salt-stress related *dehydrin* from the halophyte *I. pes-caprae,* named *IpDHN*, was identified and characterized to explore the possible mechanisms of *I. pes-caprae* response to extreme salt and drought stresses and to further explore the potential implication of *IpDHN* in areas of genetic breeding in improving the performance of plants under stress.

Environmental stress, especially high salt stress, is an important cause of poor plant growth and crop reduction. At present, numerous studies have focused on the functional genes that play pivotal roles in salt tolerance and have committed to elucidating the genetic and molecular bases of improving plant resistance; consequently, some truly useful or positive genetic resources for salt stress have been discovered (Ventura et al., 2015). In this study, the multifunctionality of *IpDHN* was explored based on sequence analyses, gene expression patterns, promoter isolation, transgenic overexpression assay, and other physiological and biochemical tests. This is the first report of the characterization of an abiotic stress tolerance *dehydrin* from the extreme halophyte *I. pes-caprae.*

Dehydrins are a class of high hydrophilic proteins that often responding to water-deficit stresses, such as freezing, drought, and high salinity. Sequence analysis indicated that IpDHN shares typical DHN motifs, including an S-segment and three K-segments, similar to the SK_n_-type DHNs of other plant species (Hara, 2010) (**Supplementary Figure S2B**). IpDHN showed high amino acid identity with the banana DHN MusaDHN-1 (**Supplementary Figure S2B** and **Figure 1**), which was reported as an osmotic stress-tolerance protein, and when overexpressing *MusaDHN-1* in transgenic banana plants, the plants showed superior performance under drought and salt stress conditions (Shekhawat et al., 2011). In Arabidopsis, there is a small subfamily of DHNs that belong to the LEA family (Alsheikh et al., 2005; Hara, 2010; Candat et al., 2014). Phylogenetic analysis indicated that IpDHN has close evolutionary relationships with ERD14 (At1g76180.1), COR47 (At1g20440.1), and ERD10 (At1g20450.1) (**Figure 1**). Previous studies showed that in Arabidopsis, both ERD14 and ERD10 accumulated under dehydration and salt treatments, while COR47 accumulated primarily in response to low temperature (Nylander et al., 2001; Kovacs et al., 2008). Several potential phosphorylation sites, including mainly the S-fragment and other serine/threonine sites, have been found in all three acidic DHNs (Alsheikh et al., 2005), and the phosphorylation status of DHNs *in vivo* would affect the ion-binding feature and even the biological role in plants (Riera et al., 2004; Hara, 2010). Here, in IpDHN, a typical S-segment containing 9 tandem serines was also found and implied that IpDHN has similar phosphorylation characteristics and biological functions involved in dehydration responses and ion-binding features (**Supplementary Figure S2B**). DHNs are highly hydrophilic, and in our research, analysis of amino acid constituents showed that IpDHN contained significant amounts of hydrophilic amino acids (**Supplementary Table S2**), further indicating the high hydrophily of IpDHN. The subcellular localization of IpDHN also seemed to have similar patterns to ERD14, ERD10, and COR47 based on the GFP fusion protein detection assay (**Figure 4**) (Shekhawat et al., 2011), which further implied that IpDHN had an approximate organelle distribution.

Expression profiles revealed that, similar to *ERD14*, *ERD10*, and *COR47*, *IpDHN* is expressed constitutively in all tissues (Nylander et al., 2001). In addition, *IpDHN* was significantly induced by multiple abiotic stresses, such as high salinity, dehydration, oxidative stress, and cold (**Figure 5**). Furthermore, *IpDHN* was also induced by ABA treatment, consistent with *ERD14*, *ERD10*, and *COR47,* suggesting that *IpDHN* participated in the response to abiotic stress possibly mediated by the ABA signaling pathway. The isolated promoter region of *IpDHN* contains several stress-related *cis*-elements, further revealing that the expression of *IpDHN* is responsive to multiple stresses.

It was previously reported that *dehydrins* exist widely in plants not only in halophytes or drought-endurance plants, which means that besides the biochemical features of DHN proteins *per se*, the gene expression regulation pattern might be an issue as crucial as the proteins. Halophytes have been considered to be more tolerant of abiotic stress due to their high and differential regulation patterns of the similar basic set of stress-responsive genes present among all plants (Glenn et al., 1999; Mishra and Tanna, 2017). The promoter region, especially the transcriptional regulatory elements detected by some transcription factors, should be critical entry points to understand the transcriptional regulatory mechanism and gene expression pattern. Since halophytes have highly developed biochemical tolerance mechanisms to high salinity (Glenn et al., 1999), the halophytic promoters have emerged as a promising and powerful candidate for plant genetic engineering about abiotic stress tolerance in crops for the regulable or high-level expression of transgenes (Mishra and Tanna, 2017). Recently, some studies on *cis*-elements of stress-responsive genes’ promotors from halophytes have been reported. A putative promoter region (1,023 bp) of the *SbGSTU* gene from *Salicornia brachiata* showed salinity and dehydration-induced activity in transgenic tobacco (Tiwari et al., 2016). The promoter of the *AlSAP* gene from the halophyte grass *Aeluropus littoralis* also showed a stress-inducible expression pattern in transgenic rice (Ben-Saad et al., 2015). A promoter region of the SNARE gene (*SbSLSP*) from *S. brachiata* was cloned, and sequence analyses indicated it had several *cis*-regulatory motifs related to abiotic stress; the expression of *SbSLSP* seemed to be up-regulated upon salinity and dehydration stresses (Singh et al., 2016). In the current study, the cloned *IpDHN* promoter region (974 bp) contained several potential *cis*-acting elements that respond to environmental stress, such as ABRE, MBS, and TC-rich repeats (**Figure 2**). In addition, the expression of *IpDHN* in *I. pes-caprae* was also regulated by abiotic stresses and ABA (**Figure 5**). The ABA responsive element (ABRE) is a key *cis* element in ABA signaling (Hobo et al., 1999), and has been reported broadly involving in ABA signaling and osmotic stress (Mishra et al., 2014; Watanabe et al., 2017). Furthermore, a designed synthetic promoter containing tandem ABREs has been proved having inducible response of exogenous ABA and stresses with tissue-specific patterns, and has the potential application and contribute to developmental and environmental control of specific gene expression in specific organs (Wu et al., 2018). The myb-binding site (MBS) *cis* element has been known to be important for plant ABA signaling and stress response, especially responding to drought inducibility (Urao et al., 1993; Abe et al., 2003). Here our qRT-PCR analysis in *I. pes-caprae* seedlings indicated that *IpDHN* was dramatically induced by salt, mannitol and ABA, and we speculate that this putative *IpDHN* promoter may be a stress-responsive promoter. Considering the less obvious difference of amino acid sequence between the IpDHN (DHN from halophyte *I. pes-caprae*) and InDHN (DHN from glycophyte *Ipomoea nil*) (**Supplementary Figure S2B**), here we can conclude that the promoter region of *IpDHN* might play a bigger part in *I. pes-caprae* adapting salt/drought environment. Accordingly, this promoter’s biological features can be verified by promoter-driven *GUS* expression transgenic assay in Arabidopsis.

As single-celled organisms, yeast and *E. coli* have been used as tools to study gene functions in multicellular organisms, especially with respect to plant osmotic stress tolerance (Zhu et al., 1997; Dang et al., 2014; Saucedo et al., 2017). *IpDHN* cDNA was isolated by a cDNA library screening assay with a yeast salt sensitive mutant, AXT3 (data not published), with the purpose of characterizing the salt-tolerance molecular mechanism of *I. pes-caprae*. In our study, because of the advantages of protein induction in *E. coli*, we also checked whether the accumulation of IpDHN in *E. coli* cells could enhance the abiotic tolerance of transgenic *E. coli* strains. To date, many previous reports have focused primarily on the major groups of LEA protein (including DHNs) involved in osmotic stresses that have been examined in *E. coli* (Ling et al., 2016; Saucedo et al., 2017). Arctic *Cerastium arcticum* is a hardy plant, and Kim et al. (2013) reported that a *C. arcticum* DHN *CaDHN* gene enhanced the tolerance of yeast to oxidants, osmotic stresses, and metal toxicity. Another study conducted on the hardy plant *Prunus mume* showed that five *dehydrin* genes from *P. mume* (*PmLEA8*, *PmLEA10*, *PmLEA19*, *PmLEA20*, and *PmLEA29*) could enhance *E. coli* tolerance to salt and sorbitol stresses (Bao et al., 2017). A Y_3_SK_2_-type *dehydrin* gene from cucumber (*Cucumis sativus*), *CsLEA11*, could also enhance tolerance to high or low temperature when overexpressed in *E. coli* (Zhou et al., 2017). Our research demonstrated that GST-IpDHN can be easily and obviously induced in *E. coli* BL21 (**Figure 3A**), and the spot assay, single clone counting assay, and cell growth curves of *E. coli* showed that IpDHN could significantly enhance salt/drought and sorbitol tolerance of *E. coli* cells (**Figure 3B** and **Supplementary Figures S4A,B**).

Some reports indicated that DHNs can directly scavenge free ROS due to their high percentages of amino acid residues, such as glycine, histidine, and lysine (Dean et al., 1997; Hanin et al., 2011), or can bind to metals to prevent excessive ROS formation in intracellular compounds (Hanin et al., 2011). In a previous study, we found that *IpDHN* could partly rescue the H_2_O_2_ sensitivity of the yeast mutant strains *yap1*Δ and *skn7*Δ to H_2_O_2_, as well as elevate the salt tolerance of AXT3 and W303 (**Supplementary Figure S1**). A similar assay for improved H_2_O_2_ tolerance was also performed using the *E. coli* expression system. Herein, the accumulation of GST-IpDHN proteins obviously increased the tolerance of *E. coli* to H_2_O_2_ (**Figure 3B**), which further demonstrates that IpDHN has antioxidative ability.

Cellular ROS accumulation as a result of stress is a widespread phenomenon in plants (You and Chan, 2015), and this research provided evidence that IpDHN could be involved in ROS scavenging (**Figures 3B**, **11** and **Supplementary Figures S1, S4A**) and could thereby improve organism’s tolerance to environmental stresses, such as dehydration, high salinity, and oxidative stresses. The excessive accumulation of IpDHN in cells maintains not only the water status under stress conditions (**Figures 7**–**9**), but also directly scavenges ROS for cellular detoxification (**Figures 11A,B**). Furthermore, it can be hypothesized that IpDHN acts as a molecular chaperone to increase the activity of protective enzymes (**Figures 11C,D**) or to activate some transcriptional processes to up-regulate the expression of stress-responsive genes, including some antioxidant enzyme system genes and other stress-responsive genes (**Figures 12A,B**).

There is increasing evidence that plant DHNs can scavenge ROS generated from cellular metabolism, thereby increasing the ability to cope with abiotic stresses based on a plant transgenic heterologous overexpression assay (Liu et al., 2015; Saibi et al., 2015; Cao et al., 2017; Li et al., 2017; Halder et al., 2018). Generally, the cellular accumulation of DHNs has pleiotropic effects on increasing plant tolerance to abiotic stresses, not only by binding directly to water molecules, ROS, transition metals, membrane lipids, or cellular skeletons to maintain the basic cellular metabolism, but also by binding to proteins, such as enzymes and transcription factors, and serving as molecular chaperones to maintain the basic activities of these functional proteins, thereby producing a positive influence on cell livability under abiotic stresses. In this study, a series of transgenic assays (including yeast, *E. coli*, and plant) were performed with overexpressed *IpDHN*, and the results indicated that the cellular accumulation of IpDHN indeed improved the tolerance to salt and drought stresses, possibly by activating the oxygen scavenging system.

A physiological assessment showed that *IpDHN-*overexpressed Arabidopsis plants showed better indicators than the wild-type plants, which is related to better salt/drought tolerance in transgenic Arabidopsis than in the WT plants. RWC is a relevant tool for the measurement of plant osmotic stress tolerance. The value of RWC in transgenic Arabidopsis was slightly higher than in WT when they were subjected NaCl or mannitol stresses (**Figure 10A**). Cellular accumulation of free proline is frequently observed during the challenge of plants to adverse environmental and stresses (Ben Rejeb et al., 2014). Proline acts as both an osmoprotectant and a potent non-enzymatic antioxidant, thereby maintaining cell viability and preventing oxidative damage caused by ROS. A higher accumulation of proline was measured in the *IpDHN* overexpression lines (**Figure 10B**), indicating that under osmotic stress challenges, *IpDHN* overexpression provided better protection by regulating proline metabolism to maintain the growth of plants. As a significant indicator of cellular membrane injury (Dexter et al., 1932), the IL value was significantly higher in the WT than in transgenic lines, suggesting that the transgenic plants suffered less membrane damage than the WT (**Figure 10C**). MDA is a final product of lipid peroxidation caused by ROS and is therefore a key indicator of osmotic stress injury in plants (Moore and Roberts, 1998). Thus, our research implied that lipid peroxidation caused by accumulation of ROS was somewhat alleviated in transgenic plants under osmotic stresses (**Figure 10D**).

As we can see from **Figure 11**, transgenic overexpression lines (*IpDHN OX1* and *IpDHN OX2*) accumulated a lower level of H_2_O_2_ (**Figure 11A**) and O_2_^−^ (**Figure 11B**) under drought/osmotic stress compared to the WT, which demonstrates that the ROS scavenging systems might be more effective in *IpDHN* transgenic plants than in WT. The activities of two antioxidant-related enzymes were measured in this research. In general, SOD and CAT activities were significantly higher in the transgenic than in the WT plants, and by combining the quantitative RT-PCR analyses results of *CAT1* and *CSD1* in Arabidopsis (**Figure 12A**), we can draw a preliminary conclusion that, by means of some pleiotropic effects, the overexpression of *IpDHN* in plants can significantly improve tolerance to salt/drought stresses, probably by mediating ROS scavenging, together with the regulation of gene expression and proteins or enzymes activities.

## Conclusion

In summary, here we reported the isolation and characterization of a DHN gene, *IpDHN*, from the halophyte *I. pes-caprae*. Our research showed that IpDHN belongs to an SK_3_ type DHN. Expression of *IpDHN* in *I. pes-caprae* is induced by mannitol, salt, MV, cold, and ABA. Analysis of the promoter of *IpDHN* identified *cis*-elements involved in growth, developmental processes, and a variety of stress responses. Overexpression of *IpDHN* in microorganisms and Arabidopsis displayed complex patterns involved in abiotic stresses, including mainly salt and drought tolerance. In Arabidopsis, the overexpression of *IpDHN* increased the resistance of transgenic plants to high salinity and drought probably by changing the accumulation of several physiological parameters and by promoting the expression of some key genes in the osmotic stress pathway, ROS scavenging system, and drought-responsive pathway. These findings indicated that IpDHN acts as a downstream effect factor in plant response to high salinity and drought stresses and may be a promising candidate gene for genetic breeding of salt-tolerant plants. In summary, our research confirmed the significant value of the continued investigation into the function and mechanisms of the *IpDHN* gene in *I. pes-caprae* for the further development of stress-tolerant crops in genetic breeding.

## Author Contributions

MZ, KX, and SJ conceived the study and designed the experiments. MZ, HZ, JZ, and HS performed the experiments. HZ and JZ analyzed the data with suggestions by MZ. MZ drafted and revised the manuscript. All authors read and approved the final manuscript.

## Conflict of Interest Statement

The authors declare that the research was conducted in the absence of any commercial or financial relationships that could be construed as a potential conflict of interest.

## Footnotes

1 https://www.ncbi.nlm.nih.gov/orffinder/

2 http://prosite.expasy.org/

3 http://www.sbg.bio.ic.ac.uk/phyre2/html/page.cgi?id=index

4 http://clustalw.ddbj.nig.ac.jp/

5 http://bioinformatics.psb.ugent.be/webtools/plantcare/html/

6 http://bioinformatics.psb.ugent.be/webtools/plantcare/html/

## Abbreviations

ABRE: ABA responding elements
CAT: catalase
cDNA: complementary DNA
CDS: coding cDNA sequence
DHN: dehydrin
ERD: early responsive to dehydration
GFP: green fluorescent protein
IDP: intrinsically disordered proteins
IL: ion leakage
LEA: late embryogenesis abundant
MBS: MYB binding site
MDA: malondialdehyde
MS: Murashige and Skoog
ORF: open reading frame
OX: overexpression
PCR: polymerase chain reaction
qRT-PCR: quantitative reverse transcription polymerase chain reaction
ROS: reactive oxygen species
RWC: relative water content
SOD: superoxide dismutase
WT: wild type.
