# Supplementary material for: Molecular Cloning and Functional Characterization of the Dehydrin (IpDHN) Gene From Ipomoea pes-caprae
Source: Front Plant Sci. 2018 Oct 11;9:1454. doi: 10.3389/fpls.2018.01454 (PMC6193111; doi:10.3389/fpls.2018.01454)
Supplement: Supplementary file 1 [file Data_Sheet_1.docx]

**SUPPLEMENTARY MATERIAL**

**Supplementary Data Figure S1.** **Functional analysis of IpDHN in *Saccharomyces cerevisiae* for salt and H_2_O_2_ tolerance.** (**A**) The IpDHN can partly rescue the phenotype of the salt-sensitive mutant strain, AXT3; (**B**) The growth performance of the wild type yeast W303 expressing IpDHN on SDG plates containing different concentrations of NaCl (0, 5%, 7.5%, and 8.8%); (**C**) and (**D**) The oxidative resistance test of IpDHN expressed in the H_2_O_2_-sensitive mutant yeast strains *yap1Δ* (C) and *skn7Δ* (D).

**Supplementary Data Figure S2.** **Sequence information of IpDHN.** (**A**) Nucleotide and deduced amino acid sequences of *IpDHN* cDNA. The nucleotides are numbered on the right. The start codon (ATG) and the stop codon (tag) in the frames are boxed; (**B**) Multiple sequence alignment of IpDHN with its homologous sequences. The amino acid sequences of S-segments and K-segments are highlighted with lines. InDHN (XP_019187872.1); CcDHN (ABC68275.1); CaDHN (NP_001311855.1); StDHN (AAB53203.1); (**C**) The genomic sequence of *IpDHN* (red: exon1 and exon2; green: intron).

**Supplementary Data Figure S3.** **The 3D diagram of the IpDHN protein predicted by PHYRE^2^** (<http://www.sbg.bio.ic.ac.uk/phyre2/html/page.cgi?id=index>).

**Supplementary Data Figure S4. IpDHN induced-expression for salt, drought, and H_2_O_2_ tolerance in *E. coli* by growth curve and colony counting.** (**A**) Growth kinetics of *E. coli* transformed with pGEX 6p-1 (control) and IpDHN-pGEX 6p-1. Cells were grown until an optical density of 0.5 was reached at 600 nm; then, 0.2 mM IPTG was added and incubated for 4 h at 30°C. Subsequently, the cells were transferred to fresh LB medium (1:100, plus 0.2 mM IPTG) supplied with different concentrations of NaCl (3% or 4%), sorbitol (0.8 M or 1M), or H_2_O_2_ (0.7 mM or 0.9 mM). The bacteria were cultured at 37°C and 200 rpm. The OD600 values were measured every two hours to evaluate the growth conditions; (**B**) Cell viability related to colony forming units (CFU) before (control) and after desiccation (40°C for 4 h). Error bars indicate the ± SD based on three replicates. Asterisks indicate significant differences from the control (Student’s t-test P values, ** p < 0.01).

**Supplementary Data Figure S5.** **Photographs of transgenic lines (*IpDHN OX1* and *IpDHN OX2*) and WT seeds germinated on MS medium or MS medium with mannitol (200 mM and 300 mM) or NaCl (150 mM) for 3 weeks.**

**Supplementary Data Table S1.** Primer sequences used in this study.

**Supplementary Data Table S2.** The amino acid composition of the IpDHN protein.

**Supplementary Data Table S3.** Regulatory elements identified in the promoter regions of the *IpDHN* gene.

**Supplementary Data Figs S1**


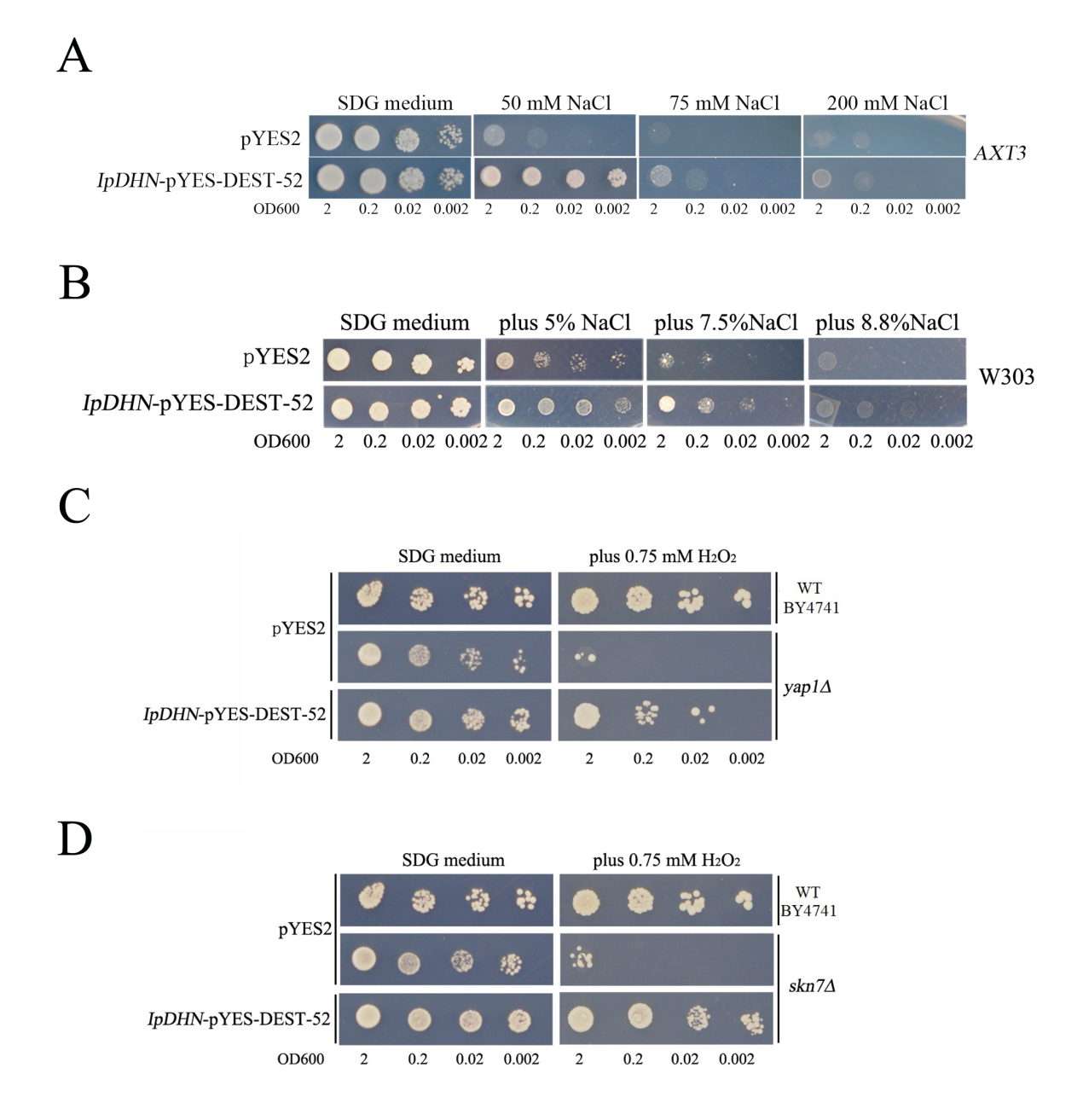


**Supplementary Data Figs S2**

**A**

**
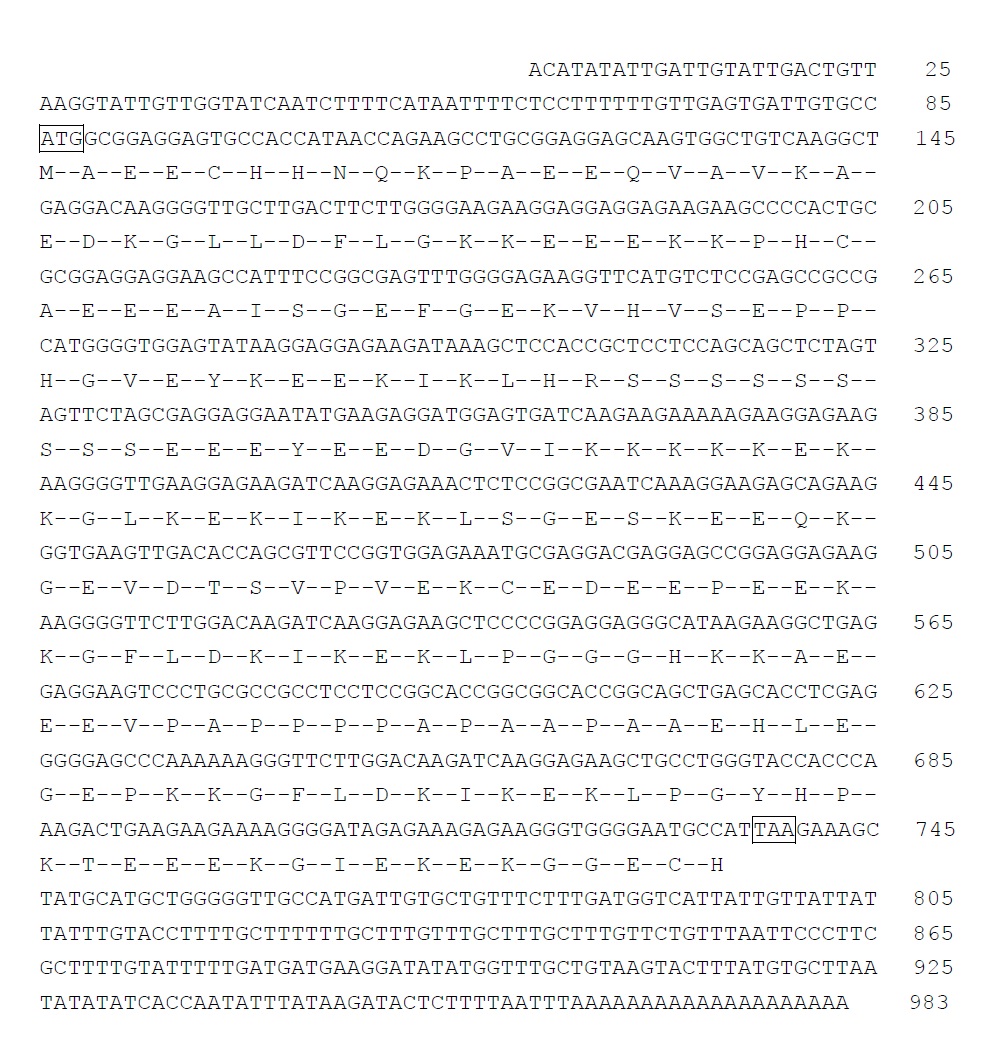
**

**B**

**
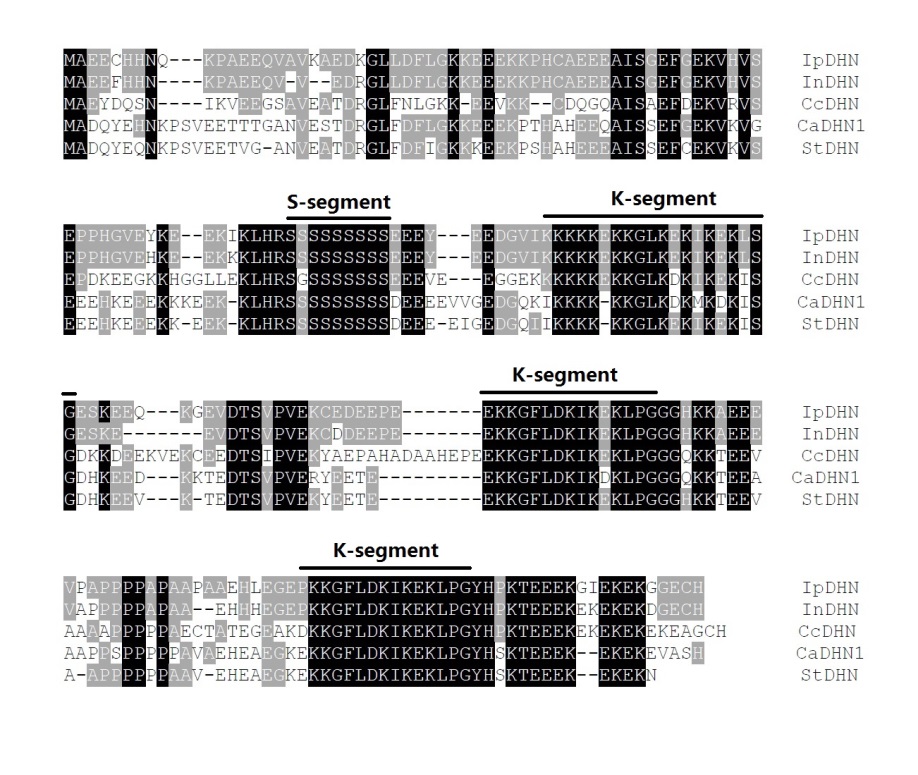
**

**C**

ATGGCGGAGGAGTGCCACCATAACCAGAAGCCTGCGGAGGAGCAAGTGGCTGTCAAGGCTGAGGACAAGGGGTTGCTTGACTTCTTGGGGAAGAAGGAGGAGGAGAAGAAGCCCCACTGCGCGGAGGAGGAAGCCATTTCCGGCGAGTTTGGGGAGAAGGTTCATGTCTCCGAGCCGCCGCATGGGGTGGAGTATAAGGAGGAGAAGATAAAGCTCCACCGCTCCTCCAGCAGCTCTAGTAGTTCTGTAAGTATTATATCCATCGATCTCTCTGTCATTTTGATGACTGATTAATCTCTTTGGATTATTTTCTTTTTGGGTTTACATCTGGTAAATCTTACATTACAATTCTAGTTGGTAAATGATTACGAATTAGTCTAGATTATTTAAAATTATTAGTGAGAGTCAAACTCCTAATCAGTCTAAATTTTATATTATTTTATAGGCTTTTTTATTGTGGTTATCAAGTCAATAATCTGTCCAGTTTGACGGGATGAACTTGTTTTGCAGAGCGAGGAGGAATATGAAGAGGATGGAGTGATCAAGAAGAAAAAGAAGGAGAAGAAGGGGTTGAAGGAGAAGATCAAGGAGAAACTCTCCGGCGAATCAAAGGAAGAGCAGAAGGGTGAAGTTGACACCAGCGTTCCGGTGGAGAAATGCGAGGACGAGGAGCCGGAGGAGAAGAAGGGGTTCTTGGACAAGATCAAGGAGAAGCTCCCCGGAGGAGGGCATAAGAAGGCTGAGGAGGAAGTCCCTGCGCCGCCTCCTCCGGCACCGGCGGCACCGGCAGCTGAGCACCTCGAGGGGGAGCCCAAAAAAGGGTTCTTGGACAAGATCAAGGAGAAGCTGCCTGGGTACCACCCAAAGACTGAAGAAGAAAAGGGGATAGAGAAAGAGAAGGGTGGGGAATGCCATTAA

**
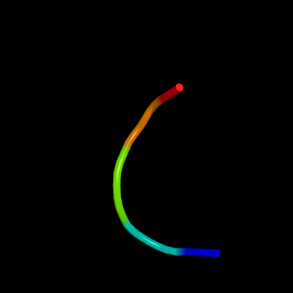
Supplementary Data Figs S3**

**Supplementary Data Figs S4**

**A**


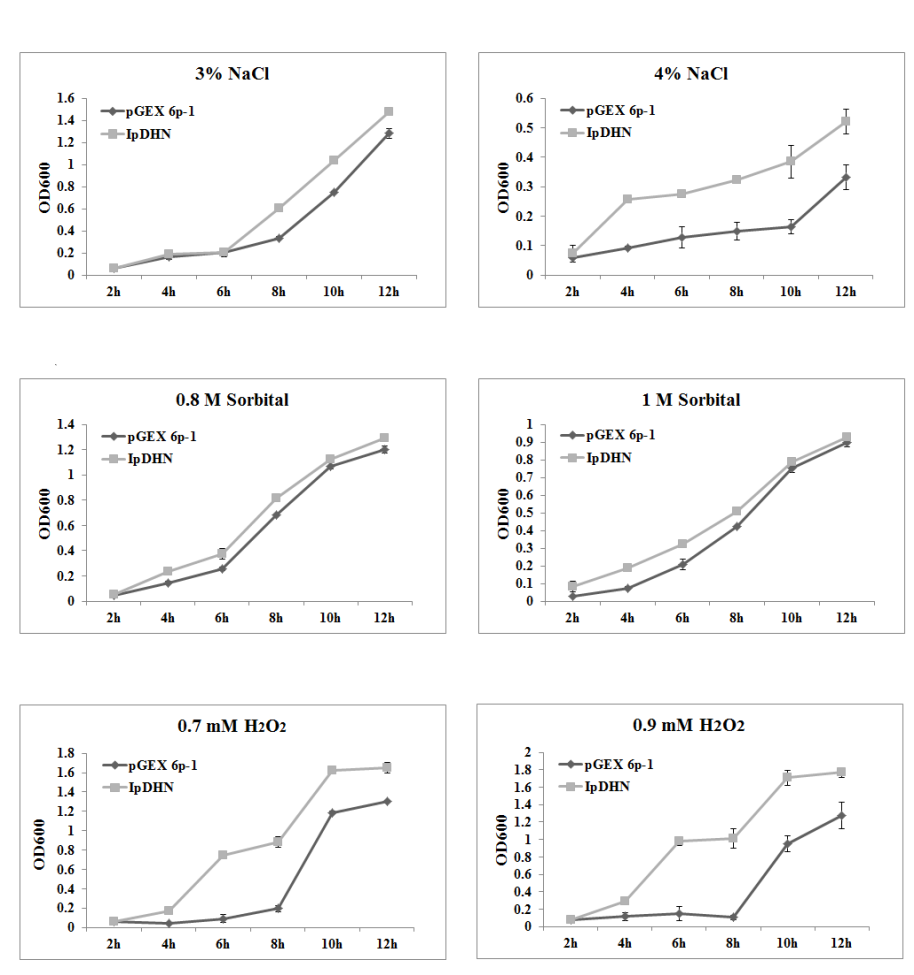


**B**


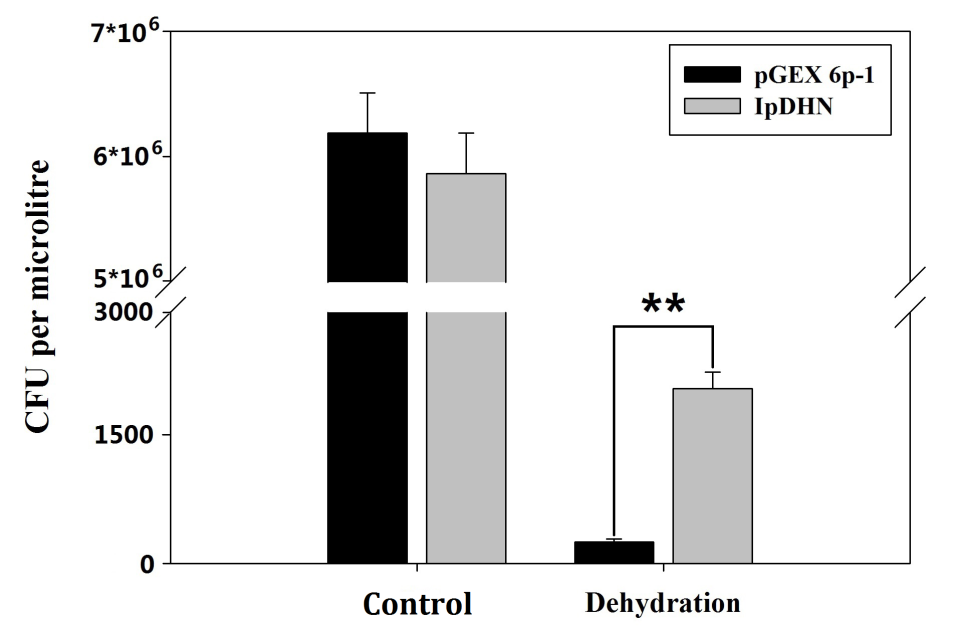


**Supplementary Data Figs S5**


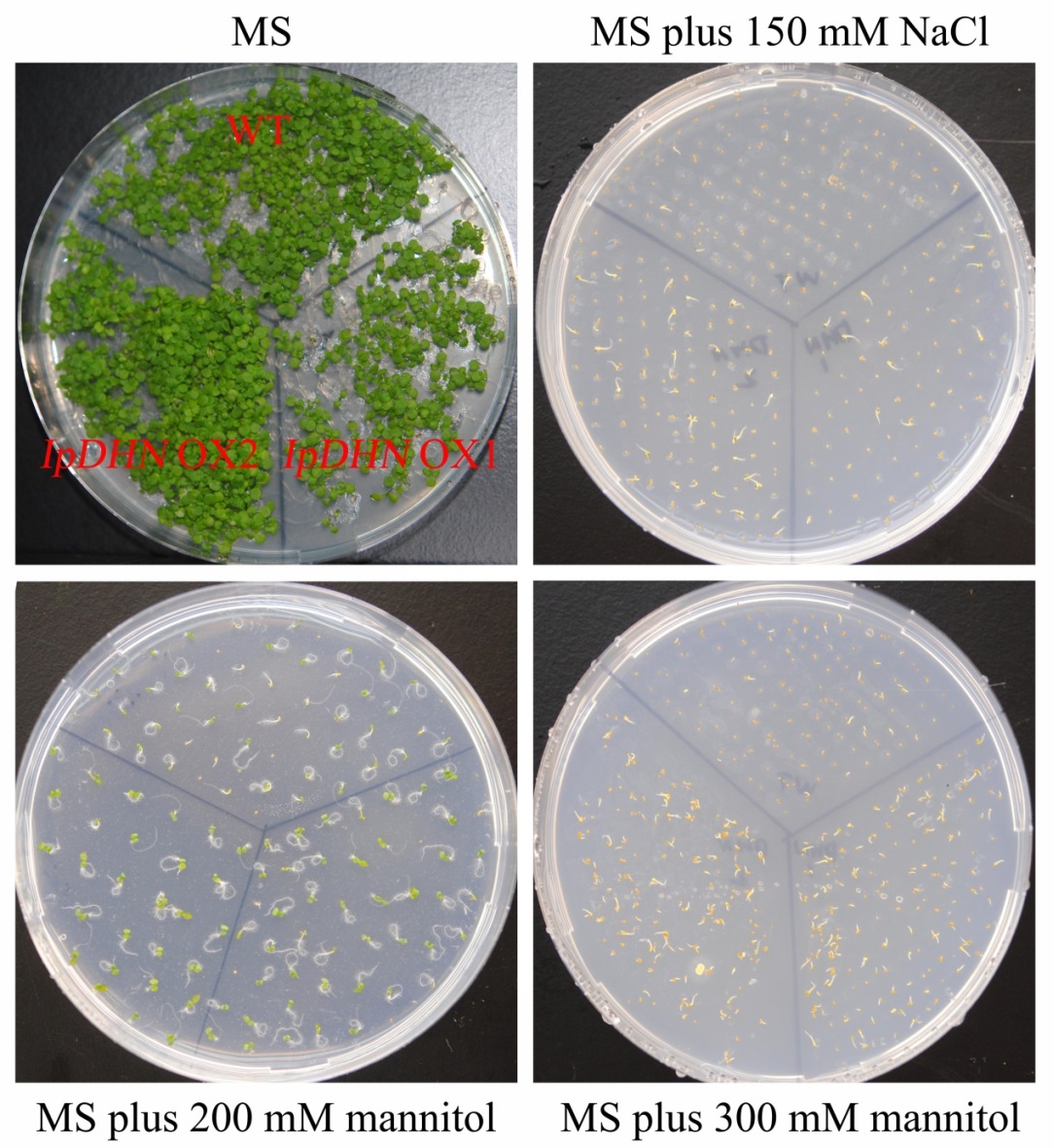


**Table S1**

| **Primer ID** | **Sequence (from 5′ to 3′)** | **Feature** |
| --- | --- | --- |
| IpDHNF | ATGGCGGAGGAGTGCCACCA | gene primer pair for sequence of *IpDHN* genomic DNA |
| IpDHNR | TTAATGGCATTCCCCACCCT |  |
| IpDHNSP1 | TCCTTATACTCCACCCCATG | IpDHN gene-specific primers for genome walking to amplify promoter sequence of *IpDHN* |
| IpDHNSP2 | GCAGTGGGGCTTCTTCTCCT |  |
| IpDHNSP3 | TGCTCCTCCGCAGGCTTCTG |  |
| IpDHNPEF | GGGGCCCCTGGGATCC ATGGCGGAGGAGTGCCACCA | gene primer pair for construction of IpDHN-pGEX 6p-1, *Bam*HI site was underlined |
| IpDHNPER | GGAATTCCGGGGATCC TTAATGGCATTCCCCACCCT |  |
| IpDHNGF | CTTGCTCCGTGGATCCATGGCGGAGGAGTGCCACC | gene primer pair for construction of IpDHN-pUC/GFP, *Bam*HI site was underlined |
| IpDHNGR | TGCTCACCATGGATCCATGGCATTCCCCACCCTTCTC |  |
| IpDHNRTF | CCTGGGTACCACCCAAAGAC | gene primer pair for qRT-PCR of *IpDHN* in *I. pes-caprae* |
| IpDHNRTR | TAAAGTACTTACAGCAAACC |  |
| IpUBQRTF | TCGACAATGTGAAGGCAAAG | gene primer pair for qRT-PCR of reference gene *IpUBQ* in *I. pes-caprae* |
| IpUBQRTR | CTTGATCTTCTTCGGCTTGG |  |
| IpDHNOXF | GGACTCTAGAGGATCCATGGCGGAGGAGTGCCACCA | for cloning the full-length ORF of *IpDHN* and construction of IpDHN-pBIm, *Bam*HI site was underlined |
| IpDHNOXR | GTCGACCCGGGGATCCTTAATGGCATTCCCCACCC |  |
| ACT2RTF | GGTAACATTGTGCTCAGTGGTGG | gene primer pair for qRT-PCR of reference gene *AtACT2* (At3g18780) in Arabidopsis |
| ACT2RTR | AACGACCTTAATCTTCATGCTGC |  |
| DHNF | GCGGAGGAGGAAGCCATTTC | gene primer pair for qRT-PCR of *IpDHN* in Arabidopsis |
| DHNR | CTCCTCAGCCTTCTTATGCCC |  |
| CAT1RTF | CGCCATGCCGAAAAATACCC | gene primer pair for qRT-PCR of *CAT1* (At1g20630) in Arabidopsis |
| CAT1RTR | CTTGCCTGTCTGAATCCCAGGAC |  |
| FSD1RTF | GCTCGGCTCTTTCCCATTGC | gene primer pair for qRT-PCR of *FSD2* (At4g25100) in Arabidopsis |
| FSD2RTR | CAGCTTCCCAAGACACAAGATTGG |  |
| CSD1RTF | TGATGGAACTGCCACCTTCACA | gene primer pair for qRT-PCR of *CSD1* (At1g08830) in Arabidopsis |
| CSD1RTR | ATGGCCTCCCTTTCCGAGGT |  |
| APX2RTF | GGAAGCTCCGTGGTCTTATT | gene primer pair for qRT-PCR of *APX2* (AT3G09640) in Arabidopsis |
| APX2RTR | CTCCTGTCTTCGTCTTCACATC |  |
| NCED3RTF | GCTGCGGTTTCTGGGAGAT | gene primer pair for qRT-PCR of *NCED3* (At3g14440) in Arabidopsis |
| NCED3RTR | TTGAGAAGACGATAATGGCGG |  |
| HAI2RTF | ACGGGCTATGGGACGTAGTG | gene primer pair for qRT-PCR of *HAI2* (At1g07430) in Arabidopsis |
| HAI2RTR | ACACATGCGCACCATCGTA |  |
| RD29ARTF | GATATCGACAAGGATGTGCCG | gene primer pair for qRT-PCR of *RD29A* (At5g52310) in Arabidopsis |
| RD29ARTR | GTATCCAGGTCTTCCCTTCGC |  |
| RD29BRTF | AAGGAGACGCAACAAGGG | gene primer pair for qRT-PCR of *RD29B* (At5g52300) in Arabidopsis |
| RD29BRTR | ACGGTGGTGCCAAGTGAT |  |
| HVA22DRTF | CAAGGCGCAGCTTTTATCTACA | gene primer pair for qRT-PCR of *HVA22D* (At4g24960) in Arabidopsis |
| HVA22DRTR | GGACGCCGTGTTTCTTGAAC |  |
| ANAC19RTF | CAACTGTGGCTACCTGAAGACGG | gene primer pair for qRT-PCR of *ANAC19* (At1g52890) in Arabidopsis |
| ANAC19RTR | CAAACGAGTCAACACCATAACCCT |  |
| RD22RTF | TTCGGAAGAAGCGGAGATG | gene primer pair for qRT-PCR of *RD22* (At5g25610) in Arabidopsis |
| RD22RTR | CTTTGAAGGCCAAGTGGT |  |
| RD26RTF | AGTTCGATCCTTGGGATTTG | gene primer pair for qRT-PCR of *RD26* ( At4g27410) in Arabidopsis |
| RD26RTR | ACCCGTTGCTTTCCAATAAC |  |

**Table S2**

| **Amino acid** | **Number** | **Percentage (%)** |
| --- | --- | --- |
| Ala (A) | 13 | 5.99 |
| Arg (R) | 1 | 0.46 |
| Asn (N) | 1 | 0.46 |
| Asp (D) | 7 | 3.23 |
| Cys (C) | 4 | 1.84 |
| Gln (Q) | 3 | 1.38 |
| Glu (E) | 49 | 22.58 |
| Gly (G) | 19 | 8.76 |
| His (H) | 10 | 4.61 |
| Ile (I) | 7 | 3.23 |
| Leu (L) | 11 | 5.07 |
| Lys (K) | 41 | 18.89 |
| Met (M) | 1 | 0.46 |
| Phe (F) | 4 | 1.84 |
| Pro (P) | 17 | 7.83 |
| Ser (S) | 14 | 6.45 |
| Thr (T) | 2 | 0.92 |
| Trp (W) | 0 | 0 |
| Tyr (Y) | 3 | 1.38 |
| Val (V) | 10 | 4.61 |

**Table S3**

| **No** | ***cis*-Element** | **Distance from ATG** | **Core sequence (5’ to 3’)** | **Putative function** |
| --- | --- | --- | --- | --- |
| 1 | TATA-Box | -178 | TATA | Core promoter element initiating transcription (*Arabidopsis thaliana*) |
| 2 | CAAT-Box | -238 | CAAT | Commom cis-acting element in promoter and enhancer regions (*Hordeum vulgare*) |
| 3 | ABRE | -48 | TACGTG | *cis*-acting element involved in the ABA responsiveness (*Arabidopsis thaliana*) |
| 4 | MBS | -52, -720, -925 | CGGTCA/CAACTG | MYB binding site involved in drought-inducibility (*Arabidopsis thaliana*, *Zea mays*) |
| 5 | CCAAT-box | -85 | CAACGG | MYBHv1 binding site (*Hordeum vulgare*) |
| 6 | Skn-1-motif | -246 | GTCAT | *cis*-acting regulatory element required for endosperm expression (*Oryza sativa*) |
| 7 | TC-rich repeats | -764 | ATTTTCTCCA | *cis*-acting element involved in defense and stress responsiveness (*Nicotiana tabacum*) |
| 8 | TGACG-motif | -245, -257, -871, -881 | TGACG | *cis*-acting regulatory element involved in the MeJA-responsiveness (*Hordeum vulgare*) |
| 9 | AuxRR-core | -345 | GGTCCAT | *cis*-ating regulatory element involved in auxin responsiveness (*Nicotiana tabacum*) |
| 10 | TCA-element | -435 | CAGAAAAGGA | *cis*-acting element involved in SA responsiveness (*Brassica oleracea*) |
| 11 | P-box | -446 | CCTTTTG | GA-responsive element (*Oryza sativa*) |
| 12 | C-Box | -258 | CTGACGTCAG | *cis*-acting regulatory element involved in light responsiveness (*Arabidopsis thaliana*) |
| 13 | G-box | -48 | CACGTA | *cis*-acting regulatory element involved in light responsiveness (*Antirrhinum majus*) |
| 15 | circadian | -477, -561, -643 | CAANNNNATC | *cis*-acting regulatory element involved in circadian control (*Lycopersicon esculentum*) |
